# Novel WRN Helicase Inhibitors Selectively Target Microsatellite Unstable Cancer Cells

**DOI:** 10.1158/2159-8290.CD-24-0052

**Published:** 2024-04-06

**Authors:** Gabriele Picco, Yanhua Rao, Angham Al Saedi, Yang Lee, Sara F. Vieira, Shriram Bhosle, Kieron May, Carmen Herranz-Ors, Samantha J. Walker, Raynold Shenje, Cansu Dincer, Freddy Gibson, Ruby Banerjee, Zoe Hewitson, Thilo Werner, Joshua E. Cottom, Yang Peng, Nanhua Deng, Youyou Zhang, Eldridge Nartey, Leng Nickels, Philip Landis, Daniela Conticelli, Katrina McCarten, Jacob Bush, Mamta Sharma, Howard Lightfoot, David House, Emma Milford, Emma K. Grant, Michal P Glogowski, Craig D. Wagner, Marcus Bantscheff, Anna Rutkowska-Klute, Cell Model Network UK Group, Francesca Zappacosta, Jonathan Pettinger, Syd Barthorpe, H. Christian Eberl, Brian T. Jones, Jessica L. Schneck, Dennis J. Murphy, Emile E. Voest, Joshua P. Taygerly, Michael P. DeMartino, Matthew A. Coelho, Jonathan Houseley, Geeta Sharma, Benjamin Schwartz, Mathew J. Garnett

**Affiliations:** 1 Wellcome Sanger Institute, Cambridge, UK; 2 GSK, Upper Providence, PA, US 19426; 3 GSK, Stevenage, UK, SG1 2NY; 4 GSK, 69117 Heidelberg, Germany; 5 GSK, Cambridge, MA, US 02139; 6 Epigenetics Programme, Babraham Institute, Cambridge, UK; 7 Candiolo Cancer Institute, Italy; 8 IDEAYA Biosciences, South San Francisco, CA 94080; 9 Netherlands Cancer Institute, Amsterdam, the Netherlands

**Keywords:** Werner helicase, mismatch repair, MSI, synthetic lethality, targeted therapy, refractory tumors, drug discovery.

## Abstract

Microsatellite-unstable (MSI) cancers require WRN helicase to resolve replication stress due to expanded DNA (TA)_n_-dinucleotide repeats. WRN is a promising synthetic lethal target for MSI tumours, and WRN inhibitors are in development. Here, we used CRISPR-Cas9 base editing to map WRN residues critical for MSI cells, validating the helicase domain as the primary drug target. Fragment-based screening led to the development of potent and highly selective WRN helicase covalent inhibitors. These compounds selectively suppressed MSI model growth *In vitro* and *In vivo* by mimicking WRN loss, inducing DNA double-strand breaks at expanded TA-repeats and DNA damage. Assessment of biomarkers in preclinical models linked TA-repeat expansions and mismatch repair (MMR) alterations to compound activity. Efficacy was confirmed in immunotherapy-resistant organoids and patient-derived xenograft (PDX) models. The discovery of potent, selective covalent WRN inhibitors provides proof of concept for synthetic-lethal targeting of WRN in MSI cancer and tools to dissect WRN biology.

## Introduction

Microsatellite instability (MSI) characterises mismatch repair (MMR) deficient cancers and is prevalent in colorectal, endometrial, and gastric tumors, among others (1,2). Some arise from somatic MMR defects, others more rarely from germline mutations, such as those in Lynch syndrome (LS)—a genetic condition associated with MMR gene mutations. MSI tumors typically are associated with better prognosis, fewer metastases, and increased survival (3,4), likely due to distinct pathological and molecular characteristics. The profusion of neoantigens generated by their hyper-mutated profile predisposes them to immunological interventions, especially when immune checkpoints are blocked (5), a process mediated by both adaptive and innate immune responses (6). This underpins the transformative impact of immune checkpoint inhibitors (ICI) on the clinical management of MSI tumors, further advanced by neoadjuvant ICI treatments (7–9). Some MSI tumors are aggressive, and innate and acquired resistance to ICI is common, especially in the metastatic setting (10–12), underscoring the need for new therapeutic strategies.

WRN helicase is involved in maintaining genome integrity, and germline mutations cause Werner syndrome, which is characterized by premature ageing and increased cancer risk (13,14). Recent work uncovered a synthetic lethal relationship between loss of the WRN RecQ-like helicase and MSI cancers (15–18). Mechanistic dissection traced WRN dependency to the accumulation of expanded DNA TA-dinucleotide repeats in MSI cells, which formed cytotoxic DNA secondary structures requiring WRN for resolution (19).

A genetic dependency on WRN is observed in a diverse set of MSI colorectal cancer preclinical models, including those resistant to standard-of-care treatments (20). This discovery has prompted efforts to target WRN pharmacologically as a novel precision strategy against MSI tumours, alone or in combination with other therapies. Small-molecule WRN inhibitors were developed before the discovery of the WRN-MSI synthetic lethal interaction (18,21) but have limited potency and selectivity (22,23). Recently, a new WRN inhibitor was reported (24), and two more (25–27) have entered clinical trials (NCT05838768 and RO7589831), but a detailed description of their activity, particularly in pre-clinical models, has not been reported and prevents an assessment of their therapeutic potential. Thus, developing potent and selective WRN helicase inhibitors and their detailed pre-clinical evaluation remains an important next step to validate the synthetic-lethal effect of WRN in MSI tumours following pharmacological inhibition.

Here, we report the discovery of novel potent, selective covalent inhibitors which bind the WRN helicase domain and provide a comprehensive evaluation of their activity in preclinical models and potential biomarkers to refine patient stratification. These inhibitors mimic genetic WRN loss, induce DNA damage, and selectively suppress MSI cancer cell model growth, including in patient-derived organoids, tumour xenografts, and PDX models of immunotherapy-resistant disease. Our findings serve as proof-of-concept for pharmacological targeting WRN’s helicase activity, paving the way for precision therapies for MSI tumors and enhancing our understanding of WRN biology.

## Results

### Mapping of targetable domains in WRN using semi-saturating mutagenesis

**Figure 1:**
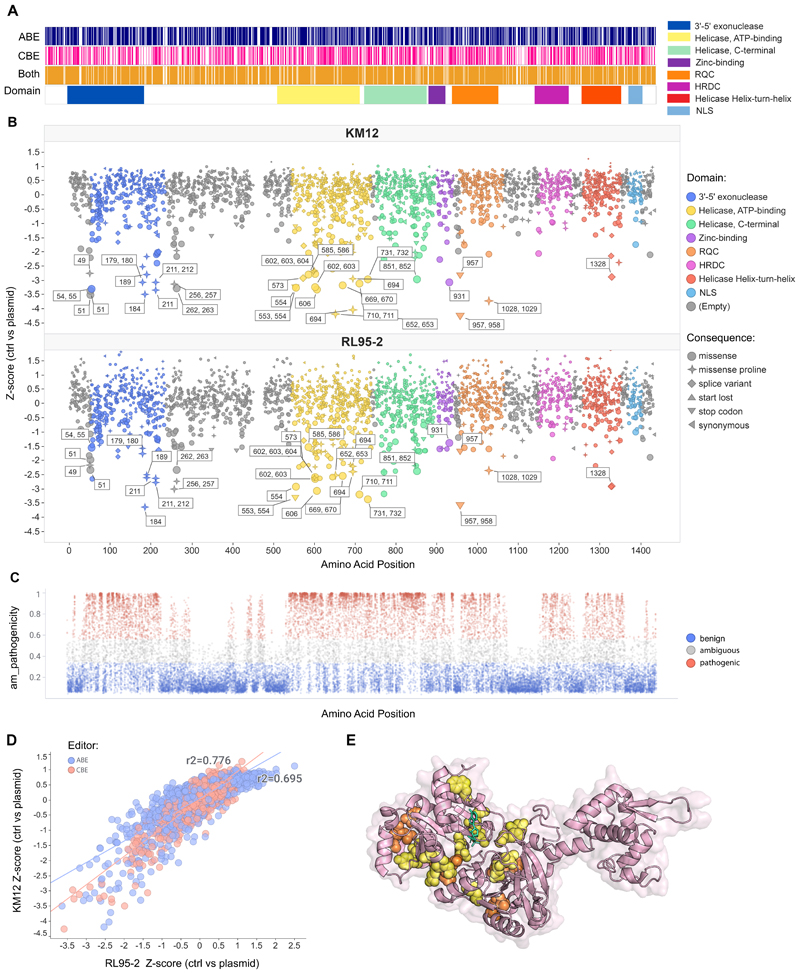
Functional interrogation of WRN domains using base editing screens in MSI cells. (A) Heatmap of predicted cytosine and adenine base editing edit sites across WRN amino acids. Individual and combined putative edits are overlaid with the main WRN protein domains. (B) Functional variant mapping of WRN for KM12 (top) and RL95-2 (bottom). Z-scores from the base editing screens for each sgRNA are displayed across WRN protein domains. sgRNAs that are referenced in the text and that introduce potential LOF and GOF positions are highlighted with their predicted edited amino acid locations. Screen z-scores for each base editor were determined individually and are shown side by side for comparison. The size of each dot in the graph is proportional to the value of the Z-score (C) Missense variants are represented as dots, plotted based on their AlphaMissense (AM) pathogenicity scores (y-axis) versus their amino acid positions (x-axis of panel B). Variants predicted to be pathogenic (red), likely benign variants (blue), and ambiguous ones (grey) were highlighted as downloaded from the AlphaMissense database (30). (D) Correlation between KM12 (y-axis) and RL95-2 (x-axis) z-scores, with ABE screens in red and CBE screens in blue. (E) The crystal structure of the WRN helicase domain (PDB ID: 6YHR) highlighting residues intolerant to variation identified by base editing screens. Missense edits predicted to introduce proline are marked in orange, while other missense variants are indicated in yellow. ATP analogue AMP-PNP in green has been mapped onto the WRN structure.

WRN has helicase and exonuclease enzymatic activity, and germline loss of function variants associated with Werner Syndrome are distributed across the gene footprint. Thus, to investigate the role of specific WRN domains in the synthetic lethal interaction with MSI, we used CRISPR base editing to introduce single-nucleotide WRN variants in two MSI cancer cell lines and assessed effects on growth. We engineered colorectal KM12 and endometrial RL95-2 cells to express adenine base editor (ABE) and cytosine base editor (CBE) doxycycline-inducible NGN base editors with high activity (28,29) (Supplementary Fig. 1A-B). Base editor cell lines were transduced with a 3735-guide library (Fig. 1A) comprising guides targeting WRN exons/promoters (n = 3107) and controls including non-targeting (n=57), intergenic (n=168), and stop codon-introducing sgRNAs for essential (n=307) and non-essential (n=87) genes (Supplementary Table 1). This mutagenesis aimed to modify >70% of WRN amino acids via cytosine and adenine base editors (Supplementary Fig. 1C). However, given variable sgRNAs efficiency, non-significant scores do not definitively exclude a residues' functional importance.

To evaluate WRN variant effects, we introduced the sgRNA library into MSI cells, induced base editor expression, and cultured cells for 10 days before quantifying sgRNAs via next-generation sequencing (NGS) (Supplementary Fig. 1D and Supplementary Table 2). We observed depletion of essential gene-targeting sgRNAs, validating known essential genes and reproducibility (Supplementary Fig. 2A-B). Combining ABE and CBE screen results showed that most sgRNAs targeting introns or predicted to cause synonymous mutations had neutral effects. In contrast, those likely causing missense mutations or splice variants were preferentially depleted (Supplementary Fig. 2C).

Our strategy identified residues required for WRN-MSI synthetic lethality, providing a map of critical WRN protein domains. We examined sgRNAs targeting the WRN coding sequence (Fig. 1B) and aligned our findings with AlphaMissense predictions (30). Regions predicted pathogenic by AlphaMissense showed an increased number of hits, thus supporting our screening approach (Fig. 1B and C). Though the exonuclease catalytic activity was considered non-essential for WRN-MSI synthetic lethality (15,16), some sgRNAs in this domain scored as hits. However, many of these edits install prolines, potentially causing protein misfolding (31). Essential residues were identified in the WRNIP1 interaction motif, which overlaps with the exonuclease domain (Supplementary Fig. 2D). The lack of vulnerability in WRNIP1 knockout MSI cells (15,32) suggests the WRNIP1 motif of WRN might have additional functions. Crucially, the helicase domain showed significant hit enrichment (Fig. 1B and Supplementary Fig. 2E), particularly in the ATP-binding subdomain, with specific residues having remarkable intolerance to variation. The comparative analysis of our screen results across both models demonstrated a uniform sgRNA depletion pattern (Fig. 1D; R2=0.77 for CBE, 0.69 for ABE), confirming the essential role of these amino acids in MSI cell viability, regardless of the model or tissue. Finally, we mapped critical sites for targeting WRN-MSI synthetic lethality on the crystal structure of the WRN helicase domain(33), highlighting residues intolerant to variation and their proximity to the catalytic center (Fig. 1E).

Overall, base editing maps of amino acid essentiality provide insights into WRN functionality, reinforcing the pivotal role of the ATP-binding helicase domain in WRN-MSI lethality, with the potential to inform the structure-guided design of WRN inhibitors.

### Reactive fragment-based screening identifies potent and selective covalent small molecule WRN helicase inhibitors

**Figure 2:**
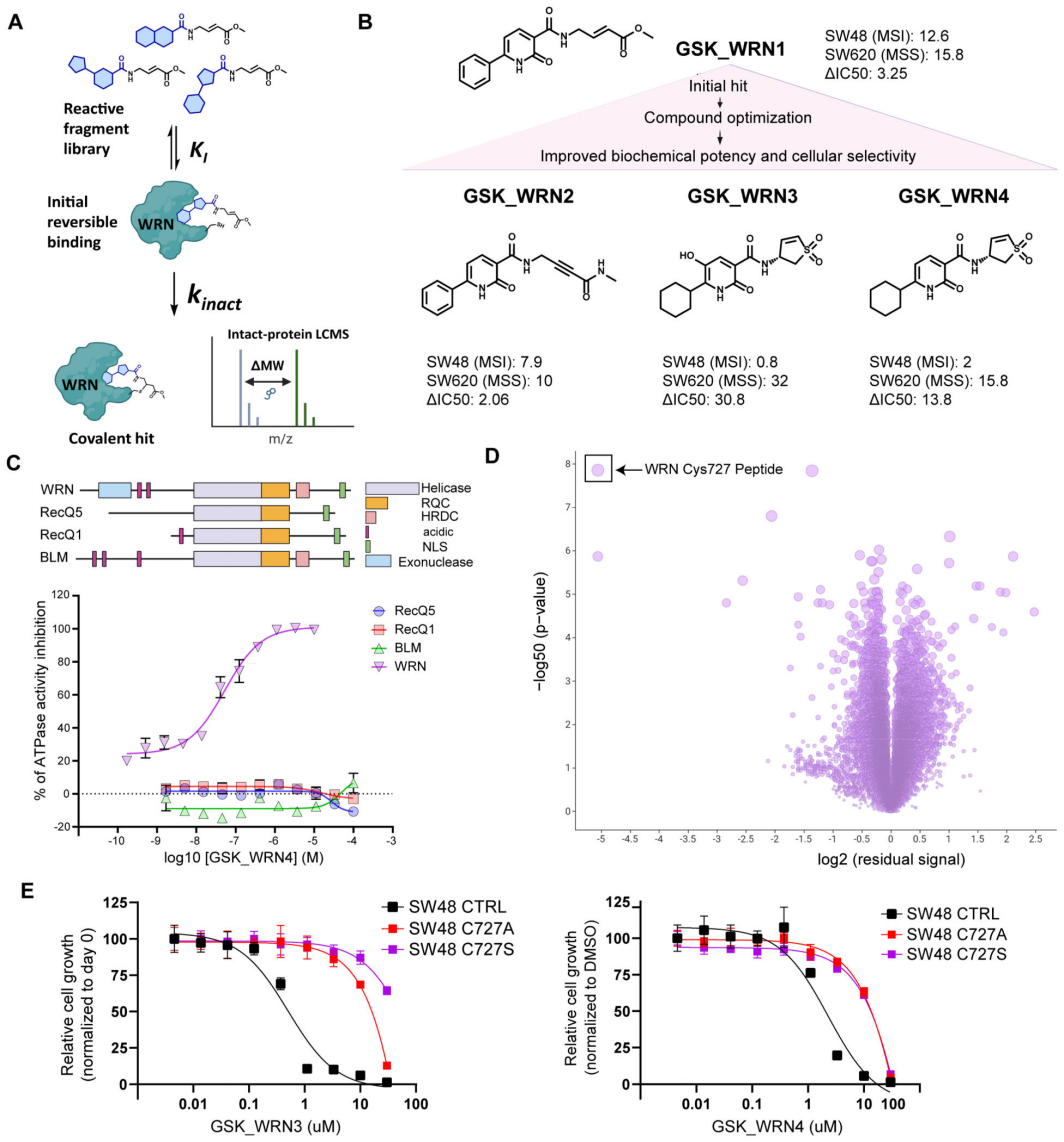
Fragment-based screening identifies potent and selective small molecule WRN helicase inhibitors. (A) Schematic overview of the fragment-based screening strategy used. (B) Structures of the WRN helicase compounds identified, from the initial hit fragment to the optimized compounds. The IC50s (μM) are reported for MSI and MSS cell lines. (C) Selectivity analysis shows GSK_WRN4 exhibits specificity towards WRN compared to other RecQ family helicases. The schematics above represent the main functional domains of RecQ DNA helicases (created with BioRender.com). (D) Volcano plot of quantitative reactive cysteine profiling of Jurkat cells treated with 10 μM GSK_WRN4. The GSK_WRN4 target cysteine residue WRN Cys727 is indicated. (E) The relative growth of SW48 control and isogenic cells (C727A and C727S) when treated with GSK_WRN3 (left panel) and GSK_WRN4 (right panel) inhibitors. Growth is normalized to DMSO controls and plotted against inhibitor concentrations. Data points reflect average values, with error bars showing the standard deviation of three technical replicates.

Base editing screens successfully pinpointed critical residues on WRN essential for its synthetic lethality in MSI cancer cells. Building on these findings, we aimed to develop inhibitors for the WRN helicase through a fragment-based screening approach (34)(Fig. 2A). Using intact-protein liquid chromatography-mass spectrometry (LCMS), we screened a library of methyl acrylate-based reactive fragments against the WRN helicase domain and identified a promising hit compound, GSK_WRN1, which gave rapid single covalent modification of WRN, achieving 81% labeling efficiency at 20 µM concentration within 24 hours at 21°C (Supplementary Fig. 3A). Tryptic digest of the WRN-GSK_WRN1 adduct and LC-MS/MS analysis revealed that the covalent hit was selectively modifying Cys727 (Supplementary Fig 3B). A focused medicinal chemistry effort was initiated to enhance biochemical potency and cellular selectivity for *in vivo* use, details of which will be presented in a subsequent report. This resulted in several compounds progressively developed from the same chemical series, including GSK_WRN2, GSK_WRN3 and GSK_WRN4 (Fig. 2B). Preliminary biochemical characterization showed that GSK_WRN3 and GSK_WRN4 significantly improved upon the inhibitory potency against WRN helicase activity compared to earlier compounds, with pIC50 values of 8.6 and 7.6 respectively, underscoring their enhanced efficacy over GSK_WRN1 (5.8) and GSK_WRN2 (6.5) (Supplementary Figure 3C).

We investigated the selectivity of this compound series. Using a fluorescence-based assay to evaluate ATPase activity (35), GSK_WRN4 had exceptional specificity for WRN over other RecQ helicases (Fig. 2C). Next, we employed a mass spectrometry-based quantitative chemoproteomic workflow (36) for cysteine-ome profiling of GSK_WRN4 in Jurkat cells. Remarkably, of 23,602 distinct cysteine-containing peptides across the proteome, WRN Cys727 was the only observed site that was almost completely modified (Fig. 2D). Even at 50 µM GSK_WRN4, few further cysteines were significantly modified demonstrating the remarkable specificity of the compound (Supplementary Fig. 3D). Introducing knock-in mutations at Cys727 rendered CRC MSI SW48 isogenic models resistant to WRN inhibition, despite having a neutral effect in the absence of the drug (Fig. 2E and Supplementary Fig. 3E).

Notably, WRN Cys727 is unique to WRN among helicase family members (Supplementary Fig. 3F), likely underscoring the selectivity observed over the other RecQ helicases.

To assess the molecular phenotype induced by these compounds, we performed a proteomics analysis using one of our early lead compounds GSK_WRN2. Treatment of the MSI cell line SW48 with 10 μM GSK_WRN2 for 48h led to a significant reduction in WRN protein (log2FC = -7.1, adjusted p-value =3.1E-6) (Supplementary Fig. 3G), consistent with reports of WRN inhibitors leading to chromatin-associated degradation (bioRxiv 2023.12.08.570895). Additionally, GSK_WRN2 induced CDKN1A (p21) and ALDH3A1, markers indicative of DNA damage and oxidative stress, respectively. Gene set enrichment analysis highlighted a reduction in genes crucial for cell cycle progression (CCNB1, NUSAP1, KIF2C), chromosome dynamics (chromatid separation and chromosome segregation and condensation)(Supplementary Fig. 3H), consistent with cellular distress due to WRN loss and consequent DNA damage and cell cycle arrest previously reported (16,17,37).

Collectively, these findings report a reactive fragment-based screening approach leading to the identification of covalent WRN helicase inhibitors with high selectivity *in vitro and in situ*.

### WRNi selectively inhibits MSI cell growth *in vitro* and phenocopies genetic inactivation

**Figure 3:**
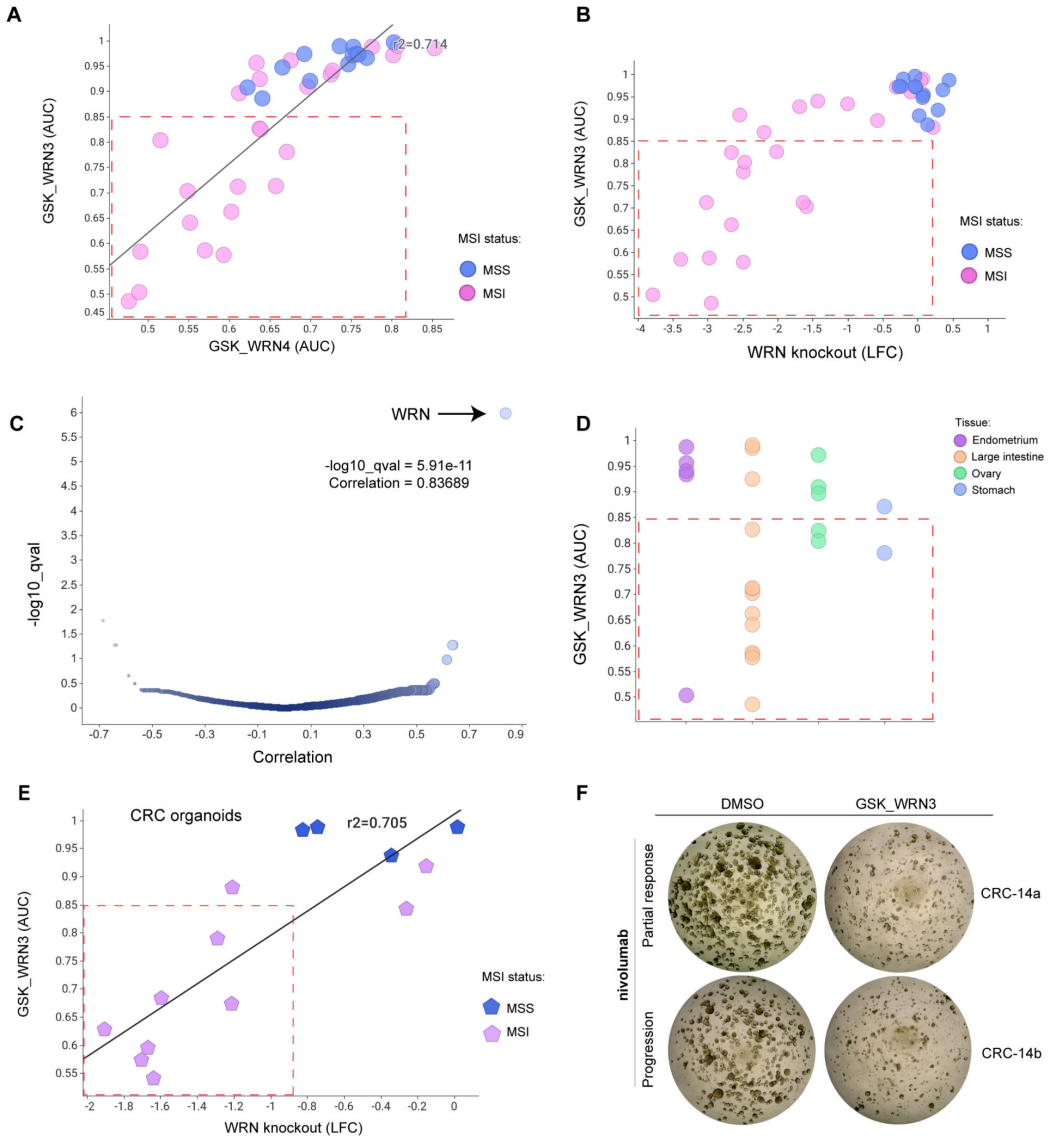
Selective Inhibition of MSI Cell Growth by WRN Inhibitors Correlates with Genetic Inactivation. (A) Sensitivity of 42 cell lines to GSK_WRN3 and GSK_WRN4. MSI cancer cell lines (pink circles) exhibit a higher level of preferential inhibition than MSS models (blue), as delineated by the red dashed box (AUC< 0.85). (B) Sensitivity of cell lines to GSK_WRN3 versus WRN CRISPR knockout log-fold change (LFC). Drug sensitivity is measured as the area under the dose response curve (AUC). (C) Genome-wide CRISPR-Cas9 gene essentiality profiles versus GSK_WRN3 sensitivity in 39 cell lines. The correlation with WRN knockout is plotted along the x-axis, while the - log10 p-value is on the y-axis. (D) The dot plot displays the AUC of GSK_WRN3 for different MSI-predominant tissues, with each dot symbolizing a cell line and colors indicating tissue type. (E) A scatter plot correlating GSK_WRN3 activity in colorectal cancer (CRC) organoids to WRN knockout LFC showcases a strong positive correlation. Color indicates MSI status. (F) Representative images of CRC organoids that are refractory to immunotherapy, treated with either DMSO or 1.25 µM GSK_WRN3. Patient clincal response to nivolumab is indicated.

To assess the potency and selectivity of the WRN inhibitors, we evaluated their effect on *in vitro* viability in curated collections of cell lines and patient-derived tumour organoids. We initially screened GSK_WRN3, GSK_WRN4, and two putative WRN inhibitors previously reported, MIRA-1 and NSC617145 (21), across 42 selected cell lines. These were enriched in MSI models from MSI-predominant lineages and part of the Genomics of Drug Sensitvity in Cancer (GDSC) collection at the Sanger Institute. We observed a strong correlation in cell line sensitivity for GSK_WRN3 and GSK_WRN4 (r2 = 0.71; Fig. 3A), and both drugs preferentially inhibited the growth of MSI cancer cell lines, while sparing MSS models. In contrast, MIRA-1 and NSC617145 displayed poor efficacy and lack of selectivity towards MSI models (Supplementary Fig. 4A). Notably, pharmacologic inhibition with GSK_WRN3 positively correlated with genetic WRN dependency by CRISPR screens in MSI-predominant lineages (r2 = 0.65 MSI only; Fig. 3B). Furthermore, unbiased correlation analysis comparing genome-wide CRISPR-Cas9 gene essentiality profiles versus GSK_WRN3 sensitivity in 39 cell lines pinpointed WRN knockout as the top hit, confirming on-target compound activity (Fig. 3C). Sensitivity to WRNi was heterogeneous across MSI-prevalent tissues, with the top sensitive models consistently from colorectal cancer (Fig. 3D).

We next evaluated the sensitivity to WRN inhibitors using an independent cohort of 15 MSI and 4 MSS CRC patient-derived organoids from the Human Cell Model Initiative (HCMI). Notably, we performed whole-genome CRISPR-Cas9 screens in 14 models to assess WRN genetic dependency, enabling direct comparison to pharmacologic WRN inhibition. In this set, sensitivity to WRNi displayed a gradient, with most models (n=7) having apparent sensitivity. In contrast, a smaller number (n=3) showed only partial sensitivity, while MSS models were fully resistant (Fig. 3E). There was a marked positive correlation between drug sensitivity and genetic knockout (r2=0.705). Furthermore, GSK_WRN3 was effective in two additional organoid models from a primary (CRC-14b) and a metastatic (CRC-14a) lesion derived from a sporadic CRC patient with a variable response to immunotherapy (Fig. 3F and Supplementary Fig. 4B). CRC-14b was derived from an immunotherapy refractory lesion and was previously characterized as WRN-addicted (20), confirmed here with pharmacological inhibition. In summary, these results demonstrate that our WRN inhibitors phenocopy the effects of genetic WRN inactivation in MSI tumours. This provides pharmacological proof-of-concept supporting WRN inhibition as a novel therapeutic approach for the treatment of patients with MSI-H cancers, including those resistant to standard-of-care therapies.

### Determinants of WRN inhibitor sensitivity in MSI cancer models

**Figure 4:**
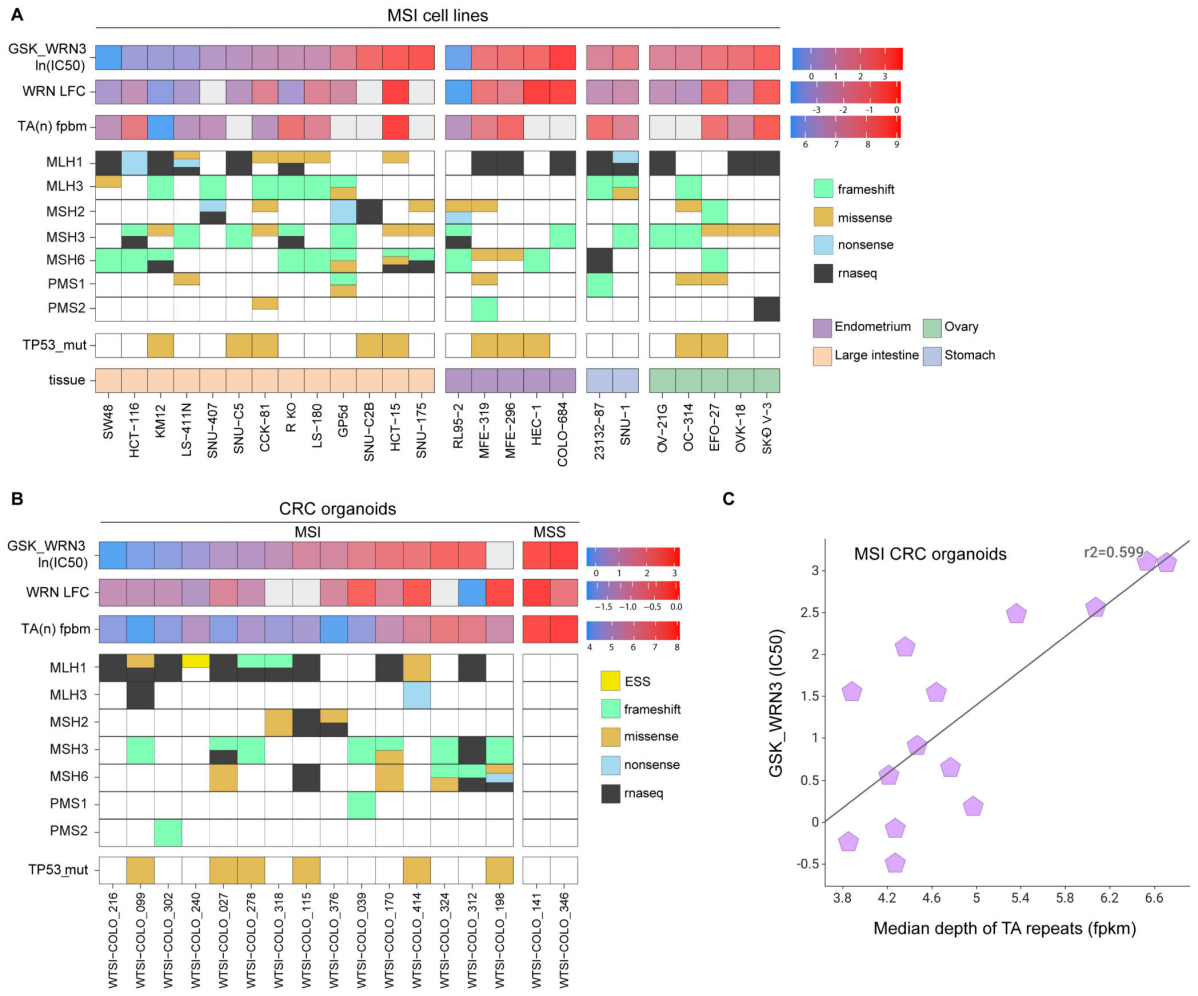
Sensitivity to WRN Inhibition in MSI Cancer Models Correlated with TA-Repeat Expansions and MMR Gene Alterations (A) Heatmap representing GSK_WRN3 sensitivity in MSI cell lines, measured by lnIC50 values. The rows illustrate data for TA-repeat expansions TA_n_ (fpbm), WRN CRISPR dependency log fold change (WRN LFC), and TP53 mutations (TP53_mut). The mutation status of MMR-pathway genes (MLH1, MLH3, MSH2, MSH3, MSH6, PMS1, PMS2) is displayed using colour-coded squares for different mutation types: exon splice (yellow), frameshift (green), missense (orange), nonsense (cyan), and RNAseq confirmation (black). The tissue origin of each cell line is shown. (B) Comparative heatmap for CRC organoids, segregating MSI and MSS profiles. The layout is similar to A, with data for two MSS models included for comparison. (C) Correlation of the IC50 of GSK_WRN3 in colorectal cancer (CRC) organoids to the median depth of 'broken' TA-repeats, as determined by coverage analysis from whole genome sequencing.

Taking advantage of our large cohorts of cell lines and organoids, we conducted comprehensive multi-omics analyses on all models to evaluate molecular factors influencing WRN inhibitor sensitivity. This included estimating TA-repeat expansions through whole genome sequencing (19,20) and assessing TP53 mutational status and alterations in MMR-pathway genes, as all have been proposed as biomarkers of WRN inhibition sensitivity (20,38). In cell lines from MSI-predominant lineages, GSK_WRN3 sensitivity correlated positively with expanded TA-repeats (r2 = 0.63 for all models and 0.56 for MSI-only models) (Fig. 4A and Supplementary Fig. 4C and D). Notably, in CRC organoids, we observed a marked positive correlation between the extent of TA-repeat expansions and GSK_WRN3 IC50 values (r2 = 0.72 for all models and 0.6 for MSI-only models; Fig. 4B and C). Many sensitive cell lines and organoids have mutations in TP53, indicating the poor predictive value of TP53 mutation as a negative biomarker of sensitivity (Fig. 4A and B). Similarly, WRNi sensitivity does not correlate with Nutlin-3a sensitivity (active only in TP53 wild-type cells) in cell lines or organoids (r2 = 0.1 and 0.02, respectively; Supplementary Fig. 4E and F). As previously reported, alterations in MLH1 showed a trend toward heightened sensitivity in CRC cell lines and organoids (20). Additionally, an independent cohort of 15 CRC MSI organoids underwent GSK_WRN4 sensitivity profiling, VENTANA IHC, WES, and RNAseq analysis, revealing only a modest association of MMR alterations with WRN inhibition (Supplementary Fig. 4G).

In summary, these results offer preclinical evidence for TA-repeat expansions as biomarkers for improved stratification of MSI patients suitable for treatment with WRN inhibitors.

### WRN pharmacological inhibition induces cytotoxic chromosomal instability and DNA damage in MSI cancer cells

**Figure 5:**
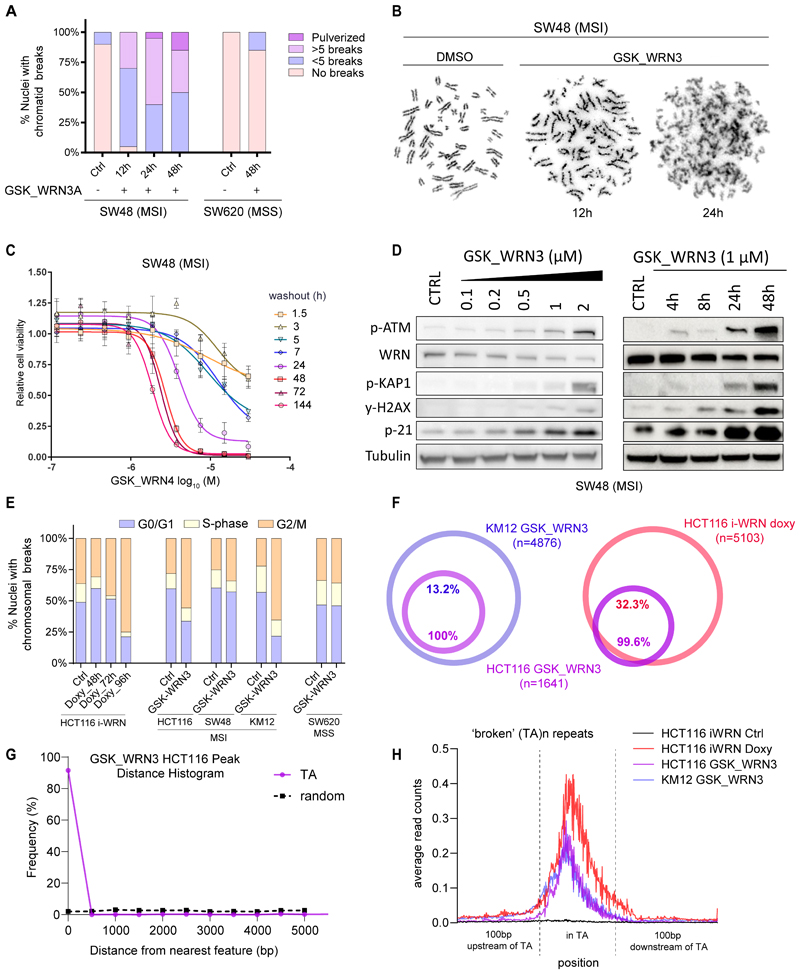
Chromosomal Instability and DNA Damage in MSI Cells Induced by WRN Pharmacological Inhibition. (A) Chromatid breaks in SW48 (MSI) and SW620 (MSS) cell lines after treatment with DMSO (ctrl) or GSK_WRN3 (2 μM). Twenty metaphase spreads were analyzed per treatment. (B) Representative images of SW48 metaphase spreads harvested after treatment with DMSO (12h) or GSK_WRN3 (12 and 24 h). (C) Time- and dose-dependent inhibition of SW48 cell growth by GSK_WRN4. SW48 cells were treated with 0.1-20 μM for 1.5, 6, 12, 24, 48, 72, or 144 hours. GSK_WRN4 was then washed out, and cell growth was assessed by cell counting over 72 hours. Data points represent the mean ± SD of three independent experiments. (D) Immunoblots of phospho-ATM, WRN, γ-H2AX, phospho-KAP1, and p21 in SW48 cells. Left panel: Post 48h treatment with GSK_WRN3, multiple concentrations as indicated. Right panel: Various time points post-treatment with 1μM GSK_WRN3 (E). Cell cycle phase distribution in HCT116 iWRN cells treated with doxycycline, and HCT116, SW48, and KM12 cells were treated with 2μM GSK_WRN3 for 24h. Data are representative of three independent experiments. (F) The overlap of TrAEL-seq peaks in HCT116 (MSI) cells treated with GSK_WRN3, KM12 cells treated with GSK_WRN3, and HCT116 cells with CRISPR-mediated WRN knockout (i-WRN doxy). (G) Frequency distribution showing the distance in base pairs (bp) of TrAEL-seq peaks in the GSK_WRN3-treated HCT116 cells from the nearest of 67,186 annotated TA-repeat tracts (magenta line) and from 70,000 random sites (dashed line). The x-axis is capped at 5kb to emphasize the initial genomic spacing. (H) TrAEL-seq signal metaplots in HCT116 iWRN cells. The figure compares signal variations across 'broken' TA-repeats (van Wietmarschen et al. 2020)(19) under different conditions: DMSO (control), doxycycline (inducing sgRNA), and GSK_WRN3 in HCT116 and KM12 cells. The Y-axis shows average peak read counts, with the X-axis depicting relative peak positions: 100bp upstream, within, and 100bp downstream of TA-repeats.

Genetic WRN inactivation results in chromosomal defects in MSI cancer cells (16,17,20). Therefore, we investigated the effects of treating MSI cells with GSK_WRN3 by analyzing the induction of DNA damage by karyotyping. We detected the presence of structural chromosomal aberrations selectively in MSI cells within 12 hours of treatment, characterized by a striking increase over time in the number of pulverized metaphases (Fig. 5A-B and Supplementary Fig. 5A). Subsequently, compound washout experiments were conducted, revealing that a 24-hour exposure to WRN inhibitor was sufficient to inhibit completely the growth of the SW48 cell line (Fig. 5C). In contrast, the MSS SW620 cell line remained largely unaffected (Supplementary Fig. 5B). GSK_WRN3, in a dose- and time-dependent manner, replicated the effects of genetic WRN inactivation by selectively degrading WRN and concurrently upregulating DNA damage response markers such as p-ATM, p-KAP1, p21, and γ-H2AX (Fig. 5D). Moreover, GSK_WRN3 induced G2 cell cycle arrest in MSI cells (Fig. 5E). Importantly, these effects were not observed in MSS SW620 control cells (Fig. 5E and Supplementary Fig. 5C).

WRN is required to resolve DNA secondary structures at expanded TA-repeats in dMMR cells. To identify the genomic sites of DNA double-strand breaks (DSBs) in MSI cells treated with WRN inhibitors, we employed Transferase-Activated End Ligation sequencing (TrAEL-seq) (39). This method captures single-stranded DNA 3′ ends genome-wide and with base pair resolution. We used HCT116 cells engineered with an inducible sgRNA system for WRN targeting (i-WRN) as a positive control (15). Overall, pharmacological inhibition of WRN and genetic loss of WRN showed strong concordance in DSB induction across cell lines. All of the DSB peaks (n=1641) observed in GSK_WRN3-treated HCT116 cells were fully recapitulated in GSK_WRN3-treated KM12 cells, while the 99.6% (n=1637) were detected in HCT116 i-WRN cells (Fig. 5F). In the GSK_WRN3-treated and i-WRN cells, nearly all the damage peaks were located in regions with TA-repeats (Fig. 5G). We observed a marked increase in TrAEL-seq signal intensity within 'broken' TA-dinucleotide expansion regions (19) (Fig. 5H and Supplementary Fig. 5D), consistent with both WRN loss and pharmacological inhibition inducing breaks at these regions.

Our data demonstrate that pharmacological inhibition of WRN rapidly triggers hallmarks of DNA damage and chromosomal and cell cycle defects in MSI cells, and this is mediated through inducing DNA DSBs at expanded TA-repeats, closely paralleling genetic WRN inhibition.

### WRNi potently inhibits the growth of MSI models *in vivo*, including patient-derived xenografts refractory to immunotherapy

**Figure 6:**
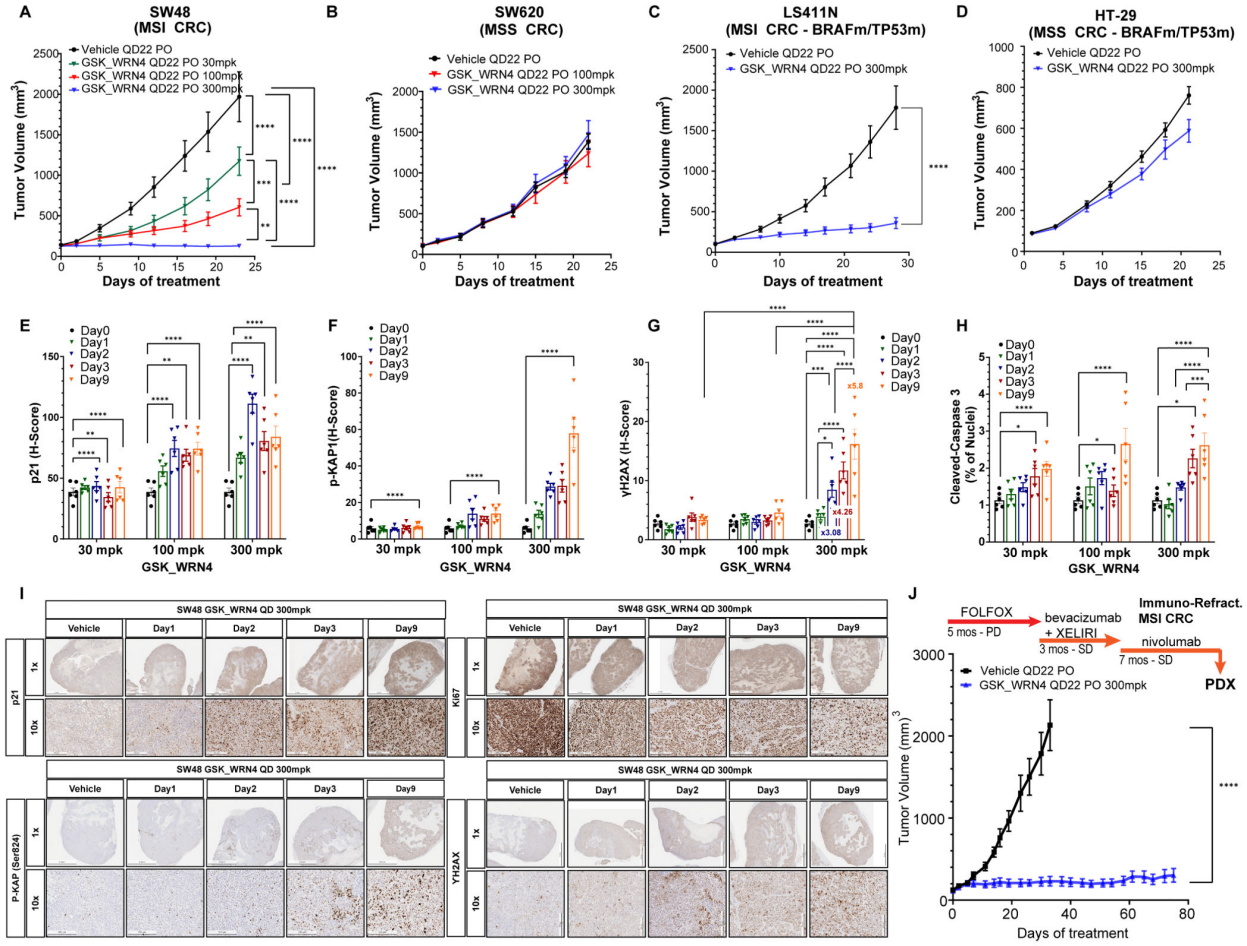
Effects of GSK_WRN4 treatment on CRC cell lines and patient-derived xenografts in vivo. (A-D) Tumor volume measurements over time for xenograft of SW48, SW620, LS411N, and HT-29 CRC cell lines treated with vehicle or GSK_WRN4 at different doses (30, 100, 300 mpk). (E-H) Quantitative analysis of p21, p-KAP1, ɣH2AX and cleaved-Caspase 3 expression levels at days 0, 1, 2, and 9 post-treatment with GSK_WRN4, presented as H-score or percentage of positive cells. (I) Representative immunohistochemical staining for p21, p-KAP1, Y-H2AX, and Ki67 in SW48 tumors treated with vehicle or GSK_WRN4 (300 mpk) at various time points. (J) Tumor volume measurements in an immunotherapy-refractory tumor xenograft derived from MSI CRC, treated with vehicle or GSK_WRN4 (300 mpk). Arrows denote the schematic timeline of clinical treatments, duration, and the corresponding tumor response before PDX establishment. *Note: Error bars represent SEM. Asterisks denote statistical significance compared to vehicle treatment (* p<0.05, ** p<0.01, *** p<0.001, ***p<0.0001). mpk: milligrams per kilogram, the unit for the administered dose. H-score: a combined score of staining intensity and percentage of positive cells. PD: Progressive disease, SD: Stable disease. PO: per os indicating oral administration QD: per daily,

To evaluate the anti-tumor efficacy of GSK_WRN4 *in vivo*, we established cell line xenografts from the MSI colorectal cancer cell line SW48 and the MSS colorectal cancer cell line SW620. Treatment with GSK_WRN4 via oral delivery led to dose-dependent tumor growth inhibition in the MSI SW48 xenografts, with tumor growth completely inhibited at the highest dosage, supported by favorable pharmacokinetics (Fig. 6A and Supplementary Fig. 6A). In contrast, GSK_WRN4 did not affect SW620 MSS xenograft growth, demonstrating high selectivity for MSI models (Fig. 6B). In addition, efficacy and selectivity were confirmed in xenografts from the LS411N (MSI) and HT-29 (MSS) cell lines, carrying TP53 and BRAF V600E mutations (Fig. 6 C and D). Mice treated with GSK_WRN4 did not display significant body weight loss, even at the highest dose, reflecting a tolerable toxicity profile (Supplementary Fig. 6B).

To investigate the on-target pharmacodynamic effects of GSK_WRN4, we analyzed the modulation of DNA damage markers (p21, phospho-gamma H2AX, and p-KAP1) in the MSI xenografts. MSI xenografts treated with GSK_WRN4 had marked dose-dependent induction of all three markers, showing that GSK_WRN4 engages the intended molecular target *in vivo* and induces DNA damage (Fig. 6E-H, 6I, and Supplementary Fig. 6C). Moreover, we found reduced proliferation marker KI67 and increased caspase-3 staining, indicating GSK_WRN4 tumor growth inhibition and apoptosis (Fig. 6H and I). GSK_WRN4 induced DNA damage specifically in MSI-H tumors, but not other tissues, demonstrating that the in vivo tumor growth inhibition by GSK_WRN4 at 300 mpk is due to WRN helicase inhibition rather than off-target toxicity. (Supplementary Fig. 6D). No significant modulation of DNA damage markers was detected in MSS xenografts (Supplementary Fig. 6E-F).

Morphological and pathological analysis of explanted tumors demonstrated that treatment with GSK_WRN4 led to a decrease in SW48 tumor cell density, accompanied by increased stroma and extracellular matrix deposition (Supplementary Fig. 6G). Additionally, the treatment led to enlarged and elongated nuclei in the tumors, similar to genetic ablation of WRN in MSI xenografts (15).

We next evaluated the efficacy of GSK_WRN4 in a patient-derived xenograft (PDX) model from a patient with treatment-refractory MSI CRC, characterized by frameshift mutations in MSH2, MSH3, MSH6, and a pathogenic TP53 missense mutation. The patient's cancer progressed despite undergoing the FOLFOX chemotherapy regimen (a combination of Oxaliplatin, Fluorouracil, and Leucovorin), and exhibited stable disease without responding to Bevacizumab and Nivolumab (Fig. 6J). Remarkably, GSK_WRN4 treatment completely inhibited tumor growth in this immunotherapy-refractory PDX.

Together, these *in vivo* studies demonstrate that GSK_WRN4 exerts potent anti-tumor activity in MSI models while sparing MSS controls, inducing hallmarks of WRN-inhibition-mediated DNA damage. The efficacy and on-target pharmacodynamic modulation support GSK_WRN4 as a therapeutic strategy for MSI cancers, including for the treatment of chemotherapy and immunotherapy-refractory tumours.

## Discussion

The concept of synthetic lethality can be exploited to develop medicines that selectively target critical vulnerabilities in cancer cells (40). This underlies the clinical success of poly(ADP-ribose) polymerase (PARP) inhibitors for BRCA-deficient cancers (41,42). Yet, the application of synthetic lethality in clinical treatments beyond the scope of PARP inhibition remains limited. The discovery of WRN-MSI synthetic lethality represents a new opportunity for synthetic lethal-based targeted cancer therapies. This study is the first detailed report of the biochemical, cellular and *in vivo* activity of novel selective WRN helicase inhibitors and provides multiple lines of evidence to support the clinical development of WRN inhibitors.

We employed a CRISPR-Cas9 base editing platform to study the WRN-MSI synthetic lethal interaction. Key residues for survival were shared across cell lines and concentrated in the helicase domain, highlighting universal dependencies on particular WRN residues. This suggests inhibitors interfering with helicase enzymatic function could serve as broad-spectrum precision therapies, effective against diverse MSI tumors reliant on WRN.

We report the discovery and development of covalent WRN inhibitors, which have potent anti-cancer effects across various MSI models. Our inhibitors display remarkable on-target activity, demonstrating efficacy and selectivity across MSI-predominant cancer lineages. Notably, sensitivity was greatest and most frequent in CRC models, with reduced and more variable sensitivity in other MSI-predominant tumour types. MSS cells were resistant to WRN inhibition, confirming the targeted efficacy and low toxicity of these inhibitors. Efforts to evaluate additional non-colorectal preclinical models would deepen our understanding of sensitivity factors in the setting of MSI and potentially widen the efficacy of WRN inhibitors to additional cancer types.

We perform the first analysis of biomarkers of response to WRN inhibition in a large set of preclinical models. Our results do not support TP53 mutations conferring resistance to WRN inhibitors (38), which is consistent with recent genetic studies (43). Notably, we validate TA-repeat expansions as significant sensitivity indicators for WRN inhibitors in CRC models, an important indication for patient selection strategies using specific molecular or genetic markers. Although reliant on whole-genome sequencing data and needing further refinement, this biomarker could be used to refine patient stratification. We noted a positive correlation between MLH1 alterations and WRN sensitivity, although specific MMR alterations do not conclusively predict sensitivity.

There is a strong correlation between WRN genetic essentiality and sensitivity to pharmacological inhibition, highlighting the compounds' specificity and validating the utility of the cancer dependency map (44) in identifying targets that are therapeutically exploitable. In addition, WRN pharmacological inhibition effectively mimicked genetic knockouts, robustly inducing DNA damage both *in vitro* and *in vivo*. Our selective WRN inhibitors, distinct from genetic tools like CRISPR knockout or RNAi, allow for precise and tunable inhibition of WRN helicase function. In-depth *in vitro* characterization of the response dynamics indicates that pulsing with WRN inhibitors can effectively induce significant DNA damage and cytotoxicity. This understanding can inform drug administration regimens during clinical development to maximise therapeutic efficacy.

We comprehensively analysed 31 patient-derived organoids with WRN inhibitors and evaluated WRN gene dependence for 14 models. This new dataset improves our understanding of WRN essentiality, which was previously largely confined to conventional 2D cancer cell line-based dependency maps, offering deeper insights into the effectiveness of WRN-targeted drugs and the determinants of drug sensitivity in 3D patient-derived preclinical models. In addition, we thoroughly validated the efficacy of WRN inhibitors *in vivo*, demonstrating their ability to selectively halt the growth of MSI tumors without causing toxicity, as they induce DNA damage only in cancer cells. The activity of WRN inhibitors on preclinical models derived from treatment-resistant tumors, including immunotherapy, support the use of WRN inhibitors for patients with treatment-refractory disease either as monotherapy or as combination therapies (20).

In conclusion, this study represents an important step forward in the development of precision medicine for MSI cancers. By integrating genomic editing approaches with fragment-based drug development, new potent and selective pharmacological WRN helicase inhibitors targeting the WRN-MSI synthetic lethal interaction were successfully developed. These compounds consistently and effectively impaired viability across diverse MSI models, expanding therapeutic options. These findings validate pharmacological WRN inhibition as a promising precision therapy for MSI malignancies harbouring this vulnerability and support the clinical development of small molecule inhibitors targeting WRN helicase for treating patients with MSI tumors.

## Methods

### Cell models

Cell lines and organoids used in this study were curated from the Genomics of Drug Sensitivity 1000 cell line collection and are annotated in the Cell Model Passports database (https://cellmodelpassports.sanger.ac.uk/)(45). Cell lines were maintained in their original culturing conditions according to supplier guidelines. Cells were supplemented with 10% FBS, 2 mM L-glutamine, and antibiotics (100 U/mL penicillin and 100 mg/mL streptomycin) and grown at 37 °C and 5% CO2 air incubator. All cell models tested negative for *Mycoplasma* using two complementary methods, MycoAlert (Lonza) and EZ-PCR (Biological Industries). Models were profiled using a panel of 94 single nucleotide polymorphisms (Fluidigm, 96.96 Dynamic Array IFC). A minimum of 75% of SNPs required to match the reference profile for a sample to be positively authenticated In addition short tandem repeat (STR) profiles were generated and matched to those provided by the cell line repositories. Models were maintained in the lab for 36 days on average (maximum 60 days) and all experiments were conducted within this period. Source of cell lines and organoids used in this study are listed in Supplementary Table 3.

### Compounds, drug screening and dose-response curve fitting

The compounds were sourced from commercial vendors or provided by GSK. DMSO-solubilized compounds were stored at room temperature in low humidity (<12% relative humidity) and low oxygen (<2.5%) environments using storage pods (Roylan Developments). Screening of cancer cell lines and organoids available at the Sanger Institute was performed using both single treatments and combinations. Compounds were screened at twelve concentrations spanning a 2,048-fold range with a 2-fold dilution series. Cells were transferred into 384-well assay plates in 40 μl (cell lines) or 60 μl (organoids) of their respective growth medium using Multidrop Combi (Thermo Fisher Scientific) dispensers. Each model's seeding density was optimised prior to screening to ensure that each cell line was in the exponential growth phase at the end of the assay. Six cell densities were tested with a two-fold dilution step; each density was dispensed into 48 wells of a single 384-well assay plate and incubated for 96 h. Cell number was quantified using CellTiter-Glo 2.0 (Promega). The maximum density tested was 3200 cells per well. Assay plates were incubated at 37 °C in a humidified atmosphere at 5% CO2 for 24 hours and then dosed with the test compounds using an Echo555 (Labcyte). The final DMSO concentration was typically 0.1%. The assay plates were incubated after dosing with compounds, and the drug treatment duration was 72 h. CellTiter-Glo 2.0 (Promega) was added, 13.5 μl for cell lines or 20 μl for organoids, to measure cell viability. Each assay plate was incubated at room temperature for 10 min before quantification of luminescence using a Paradigm (Molecular Devices) plate reader. To estimate cell growth throughout drug treatment, an additional undrugged control plate was generated, and cell viability was measured at the time of drug treatment. These plates are referred to as a ‘day = 1’ and were repeated each time a cell line was screened. All screening plates contained negative control wells (untreated wells, n = 6; DMSO-treated wells, n = 62) and positive control wells (medium-only wells, n = 12; Staurosporine-treated wells, n = 8; and MG-132 treated wells, n = 8) distributed across the plates. These control wells were used to evaluate defined quality control criteria, including the coefficient of variation (CV) and Z-factor calculated as previously described(46). A maximum threshold of 0.23 was applied to the coefficient of variation (CV), and Z-factors were required to exceed a minimum threshold of 0.3. Where a cell line was sensitive to both positive controls, it had to pass Z-factor thresholds for both positive controls. Plates that did not meet these requirements were excluded from the study.

Luminescence readings were converted to cell viabilities by normalizing with reference to the DMSO-treated wells and the positive controls (viabilities of 1 and 0 respectively). Dose response curves were then fitted to the drug-treated wells using a non-linear mixed effect model (47) to obtain IC50 estimates. Curves with a root mean squared error of greater than 0.3 were excluded from further analysis.

### Organoid culture techniques, CRISPR/Cas9 genome screening, and drug efficacy testing

The maintenance of organoids and transduction of a genome-wide single-guide RNA (sgRNA) library were performed, adapted from previously reported protocols (15,20,48,49). For drug efficacy testing of the CRC-14a a n b models, organoids were dissociated into single cells and seeded into each well of a 96-well plate with a 5 µL droplet of 80% BME solution, overlaid with organoid media. Approximately 1.5–2.5 × 10^3^ cells per well were cultured. The next day, variable concentrations of the relevant drug were added in triplicate for each model. Cell growth was monitored over 7-10 days, followed imaging with EVOS Cell Imaging System (Thermo Fisher Scientific).

### Cell line engineering for base editing

We introduced base editing machinery into cells by co-transfecting with FuGENE HD (Promega), using a plasmid encoding Cas9 and a gRNA targeting the CLYBL locus (5′-ATGTTGGAAGGATGAGGAAA-3′), alongside a plasmid with tet-ON base editor, blasticidin resistance, and mApple expression cassettes within CLYBL homology arms as recently reported (31). To enhance homologous recombination (HR) rates, cells were pre-incubated overnight with 1 μM DNA-PK inhibitor AZD7648. Transfected cells were selected using 10 μg/mL blasticidin (Thermo Fisher Scientific) for four days, followed by 5 μg/mL maintenance. Cell pools were further refined via FACS based on mApple expression. Base editing efficiency was evaluated using BE-FLARE (50). For detailed screening, clonal lines of BE3.9max NGN and ABE8e NGN were utilized.

### CRISPR-Cas9 base editing screens

Our base editing cell lines were established following the method outlined in a previous study (31). In brief, we utilized CRISPR-Cas9 and homology-directed repair to insert doxycycline-inducible CBE or ABE NGN base editors at the CLYBL safe-harbour site. Base editing cell populations were selected using blasticidin and mApple (FACS). Base editing activity was assessed using BE-FLARE (for CBE) or a GFP stop codon reporter (for ABE). We conducted base editing screens with gRNA representation of 1000-fold, utilizing viral doses that resulted in 30–50% cell infection. Post-infection, cells were selected with puromycin for 4 days. Five days post-infection, a baseline (T0) pellet was collected. Base editing was induced by administering doxycycline (1 μg/mL) for 3 days. This was followed by a 10-day selection phase, during which cells were maintained in culture (a minimum of 15 million cells in 3 Layers flask) and split regularly. Each screen was independently replicated twice on separate days for consistency. For DNA analysis, cell pellets were processed to extract DNA, and the gRNA cassette was amplified through PCR. This was done in parallel to maintain library diversity. Post column purification, PCR products were indexed and sequenced on the Illumina HiSeq2500, using 19 bp single-end sequencing and a custom primer.

### Guide RNA library and screen data analysis

To develop control guide RNAs (gRNAs) for base editing, we employed SpliceR(51), selecting the top three gRNAs using a unique metric: the product of the cDNA disruption score and the sum of ABEscore and CBEscore. For gRNAs targeting WRN exonic regions, the BEstimate tool (currently in preparation for submission and available at https://github.com/CansuDincer/BEstimate) was employed. Oligonucleotides, designed and obtained from Twist Biosciences, underwent PCR amplification. Subsequently, these amplified products were seamlessly integrated into the BbsI-cleaved pKLV2-U6gRNA(BbsI)-ccdB-PGKpuro2ABFP-W lentiviral vector using the Gibson Assembly method provided by New England Biolabs (NEB) (49). Following assembly, the resulting libraries underwent ethanol precipitation for condensation and were introduced into Endura electrocompetent cells (Lucigen) via electroporation. This step was meticulously performed in parallel to ensure the consistency of the library. The transformed cells were cultivated in LB medium supplemented with 100 µg/ml ampicillin at 30 °C grown overnight in the shaking incubator. Lentiviral vectors, along with packaging plasmids psPax2 and pMD2.G (Addgene), were transfected into 80% confluent HEK293 cells using Lipofectamine LTX (Gibco) at the specified ratio: 7.5 μg of lentiviral vector (library plasmid DNA), 18.5 μg of psPax2, and 4 μg of pMD2.G per 15 cm dish. Viral particles were harvested from the media's supernatant 72 hours post-transfection, subsequently filtered, and stored in a frozen state.

In analyzing screen results and annotating guide RNAs (gRNAs), we employed BEstimate. We assumed a wild-type (WT) genome as the baseline, disregarding specific SNPs or SNVs in the two cancer cell lines. We normalized read counts to reads per million (RPM), adding a pseudo-count for calculation purposes. We averaged RPM values from replicates for consistent gRNA analysis. Average log2fold-change (L2FC) values were calculated and z-scores were derived by normalizing L2FC values against their standard deviation. RNAs with fewer than 100 reads in initial samples were also discarded. WRN Syndrome registry variants were obtained from Friedrich et al., 2010 (52) and the Werner Syndrome Mutational Database https://www.pathology.washington.edu/research/werner/database/. Alphamissense pathogenicty score and class was downloaded from the Alphamissense manuscript supplementary informations (30). Clinical variants associated with WRN syndrome were downaload from the clin var database https://www.clinicalgenome.org/data-sharing/clinvar/. For both ClinVar and WRN Syndrome registry variants, only missense mutations were used for the analysis.

### WRN C727 mutation knock-in

CRISPR mediated homologous recombination was employed to introduce single amino acid substitutions to Cys727 on WRN in SW48 cells. To this end, sgRNA is designed to include crRNA 5’-**AAUCCUCAGAUCACCUGUAC-3’**. Cys727Ala and Cys727Ser were introduced using designed homologous recombinant templates: Cys727Ala (gcacttactgctactgcaagttcttcaatccgggaagacattgtacgttgcttaaatctgaga aaCccGcaAatTacGGCTacc ggttttgatcgaccaaacctgtatttagaagttaggcgaaaaacagggaatatcct); Cys727Ser (gcacttactgctactgcaagttcttcaatccgggaagacattgtacgttgcttaaatctgaga aaCccGcaAatTacGAGTacc ggttttgatcgaccaaacctgtatttagaagttaggcgaaaaacagggaatatcct). sgRNA and ssODNs were both synthesized at Integrated DNA Technologies. SW48 cell were maintained in RPM1640+10%FBS cell culture media and passaged with standard cell culture techniques. CRISPR-mediated knock-in was performed with electroporation of Cas9/sgRNA RNP mixture. Specifically, mix 2uL Cas9 protein (Integrated DNA Technologies #1081061,10ug/ul) with 0.5uL sgRNA(400uM) and incubated at room temperature for 15 minutes to allow RNP complex to assemble. Resuspend 4x10^5^ SW48 cells in 20uL electroporation buffer (prepared according to Lonza’s manual for Amaxa SF cell line 4D-nucleofector X Kit S (32 RCT), Lonza#V4XC-2032) mixed with 1uL of 100nM ALT-R electroporation enhancer (Integrated DNA Technologies, Alt-R HDR Enhancer V2). When RNP mix was ready, mix the cell suspension in electroporation buffer with RNP mix in one well of the electroporation chamber from the SF kit. Add 1uL of ssODN (100uM) to the cell/RNP mix and electroporate with K562 cell line protocol on the Amaxa 4D-nucleofector X unit. Transfer cells to fresh media containing 1uM of ALT-R electroporation enhancer V2 immediately after electroporation and culture for 24 hours. Change to fresh media 24 hours after electroporation.

Genomic DNA of electroporated cells were extracted with Zymo DNA extraction kit (Zymo quick DNA 96 kit; Zymo Research #D3012). 50ng of genomic DNA was used for PCR amplification of edited genomic region. A 50uL PCR reaction was set up per sample: 25uL 2 X Phusion Master Mix, 1uL forward and reverse primer mix (25uM each), 1.5uL DMSO, add H2O to 50uL. The primer sequences used for PCR amplification were: Forward (GACATGTTGGGAATGAATGAGC) and reverse (AAGAAATGGCTGCAGATCCTGA). PCR products were purified with Zymo PCR purification (ZR DNA sequencing clean up kit; Zymo Research #D4051). Purified PCR samples were sequenced using Sanger Sequencing service at GeneWiz. Knock-in efficiency was performed using Synthego ICE analysis tool (http://ice.synthego.com). Cells with sequencing verification of knock-in efficiency were treated with GSK_WRN3 and GSK_WRN4, with top dose of 30 uM and 1:3 dilution down to 4.6 nM. Compounds were dispensed on HP D300e dispenser. 2x10^3^ cells per well were cultured in 96-well assay plate (ThermoScientific # 136102) in 100uL volume with compound for 4 days. Cell viability was measured by adding 50uL CellTiter-Glo One Solution Assay (Promega #G8642). CTG (cell titer glo) readings were normalized to the DMSO wells, curve fitted and plotted in GraphPad Prism 9.0.

### Karyotype analysis with human multiplex FISH probes

Cells were harvested 12, 24, or 48 hours post-treatment with GSK_WRN3 or DMSO, following a modified standard protocol for metaphase preparation. In brief, cells in T150 flasks were treated with 0.1 g/mL colcemid (KaryoMax Colcemid Solution in PBS, Thermo Fisher Scientific) for 1.5 hours. TrypLE Express Enzyme (Thermo Fisher Scientific) was used for dissociating adherent cells into a single-cell suspension, which was then pelleted and resuspended in 0.56% KCl hypotonic solution for 12-14 minutes, followed by fixation with Carnoy's fixative (3:1 methanol:acetic acid). FISH analysis was performed according to previously reported methods (53). Metaphase slides were prepared, fixed in acetone (Sigma Aldrich) for 10 minutes, and baked at 62°C for 30 minutes. Slides underwent alkaline denaturation (0.5 mol/L NaOH, 1.0 mol/L NaCl) for 7.5-8 minutes, followed by washes in 1 mol/L Tris-HCl (pH 7.4) and 1× PBS. Dehydration was achieved through a 70%, 90%, and 100% ethanol series. The 24-color human multiplex FISH (M-FISH) probe mix was denatured and applied to the slides, with hybridization at 37°C for two nights. Post-hybridization washing included 2× SSC at 37°C, a stringent wash in 0.5× SSC at 75°C, and rinses in 2× SSC with 0.05% Tween-20 (VWR) and 1× PBS. Slides were mounted in Vectashield Vibrance Antifade Mounting medium with DAPI (Vector Laboratories). Imaging was done using an Axiolmager D1 microscope with appropriate filters, capturing digital images via SmartCapture software (Digital Scientific). Twenty randomly selected metaphase cells were karyotyped and analyzed, focusing on chromatid and chromosome breaks and complex rearrangements, using the SmartType Karyotyper (Digital Scientific) based on Multiplex FISH and DAPI banding patterns.

### Multi-omics analysis

Mutations in MMR pathway genes were downloaded from The Cell Model Passport or Dependency Map (DepMap) websites. Genetic analysis was performed as previously described (20). To identify which MMR pathway gene displayed altered gene or protein expression, we computed the Z-score by gene across all the cell lines (or organoids) in the respective data set and considered genes with Z-score or normalized values less than –2 to identify genes downregulated in a particular sample. WRN dependency was obtained mining essentiality data obtained from multiple sources: Project Score (https://score.depmap.sanger.ac.uk/) and Dependency Map (DepMap; https://depmap.org/portal/) websites. For TA-dinucleotide repeat expansion analysis, WGS data for cancer cell lines were downloaded from SRA study SRP186687.

### Western blot

Immunoblot was conducted to verify WRN depletion and DNA damage in cells treated with various concentrations of WRN inhibitor. For the blots, 4-8 × 10^6^ cells were cultured in 10 cm dishes and treated with GSK_WRN3 or DMSO. Cells were lysed at 4, 8, 24, 48, and 72 hours post-treatment using 100-150 µL RIPA buffer with proteinase inhibitors. The lysate concentration was quantified via the BCA Assay. Each sample, containing 20-30 µg of lysate, was subjected to SDS-PAGE on a 4-12% Bis-Tris gel (Invitrogen) and then transferred to a PVDF membrane. After blocking with 5% milk in TBST, membranes were incubated overnight with primary antibodies: anti-WRN (Cell Signalling Technologies, 4666, 1:1000), anti-P-ATM (Abcam, ab81292, 1:1000), anti-KAP1 (A300-767A.M, 1:1000), anti-P-Histone H2AX (NB100-384, 1:1000), anti-P21 (96109520, 1:1000), and anti-β-tubulin (Sigma-Aldrich, T4026, 1:5000) as a loading control. Blots were washed and incubated with an anti-Rabbit IgG HRP-linked secondary antibody (GE Healthcare, #NA931V-ECL HPR) for 1 hour at room temperature. Following another wash in TBST, the signal was amplified with Super Signal Dura for visualization. Precision All Blue Plus Protein Standards (BioRad, cat. 1610373) served as a molecular weight marker.

### Cell cycle analysis

We employed propidium iodide staining to evaluate cell viability. Cells were harvested using standard protocols and washed in Phosphate-Buffered Saline (PBS). The cell pellet was subsequently fixed in cold 70% ethanol and stored at 4°C for over one hour, with overnight fixation being optimal for enhanced data resolution. Following fixation, cells were washed twice in PBS and centrifuged at 1200 rpm for 5 minutes, after which the supernatant was discarded, taking care not to disturb the loose pellet. The cell count was determined for each treatment group before staining. For staining, 500 µl of propidium iodide (PI) solution at a concentration of 1x10^6 cells/ml was added to the cell pellet, followed by incubation for more than 1 hour at room temperature or overnight at 4°C. The PI solution was prepared with Triton X-100 at 0.1% (v/v), RNase A at a final concentration of 50 µg/ml, and PI at 25 µg/ml, topped up to 50 ml with PBS. Post-incubation, cells were analyzed by flow cytometry to determine viability based on PI uptake.

### TrAEL-seq for DSB mapping

For TrAEL-seq, 2-3 million cells were seeded in T150 flasks. Two days later, these cells were treated with GSK_WRN3 for 24h or with doxycycline for 72h. Cells were harvested and counted 24 hours post-treatment. 1-2 x 10^6^ cells per treatment were washed with PBS, centrifuged, and resuspended in 1mL of cold L Buffer (100mM EDTA pH8, 10mM Tris pH 7.5, 20mM NaCl), before embedding in agarose for DNA extraction. DNA ends were tailed with ATP and ligated to adaptors carrying in-line indexes, then pooled and processed into a single TrAEL-seq library as described(39). Libraries were sequenced on an Illumina NextSeq 500 as High Output 75 bp Single End by the Babraham Institute Next Generation Sequencing facility, then read were trimmed, deduplicated, de-multiplexed into individual libraries and mapped to GRCh38 by the Babraham Institute Bioinformatics Facility using scripts available at https://github.com/FelixKrueger/TrAEL-seq.

### Analysis of TrAEL-seq data

Mapped reads were imported into SeqMonk v1.48 (https://www.bioinformatics.babraham.ac.uk/projects/seqmonk/) and truncated to 1 nucleotide at the 5′ end, representing the last nucleotide 5′ of the strand break. No normalisation between datasets was performed as all data came from multiplexed libraries and should be quantitatively comparable. Peak finding was performed in each sample using MACS implemented in SeqMonk (macs2 --nomodel --tsize 100 --pvalue 1e-15 --keep-dup), and distances from MACS peaks to nearest TA tract or random sites calculated using Distance to Feature Quantitation. For the metaplot, strand specific read counts were performed at 1nt resolution over broken TA tracts defined in van Wietmarschen et al. 2020 (19) -/+ 100 bp using the Quantitation Trend Plot function. Final graphs were plotted using GraphPad Prism 10.1.1.

### Reactive fragment intact-protein LCMS screen

Fragment-Based Covalent Ligand Screening was performed as previously reported(34). Briefly, recombinant WRN helicase domain (500-946) was diluted to 0.5 µL in 25 mM HEPES, 50 mM NaCl, pH 7.5 buffer. Protein solution (10 µL) was added to the reactive fragment library (10 nL of 20 mM DMSO stock) in a 384-well plate. The plate was centrifuged at 1000 rpm for 1 minute then sealed and incubated at 21°C for 24 hours. After 24 hours, LCMS-grade H2O (10 µL) was added to all wells. Plate was centrifuged at 1000 rpm for 1 minute, sealed, and then transferred to LCMS plate hotel. Samples were measured using an Agilent G230B time-of-flight (ToF) accurate Mass Series mass spectrometer interfaced with an Agilent 1290 infinity II series column oven (G7116B) and an Agilent 1290 infinity II series liquid chromatography high-speed binary pump (G7120A). Protein samples were injected using an Agilent 1290 infinity II series multisampler with dual needles with a 2 µL injection volume. Chromatography was carried out on an Agilent Bio-HPLC PLRP-S (1000 Å, 5 µm x 50 mm x 1.0 mm, PL1312-1502) reverse phase HPLC column at 70°C. The sample was eluted at 0.5 mL/min using a gradient system from Solvent A (water, 0.2% (v/v) formic acid) to Solvent B (acetonitrile, 0.2% (v/v) formic acid) according to the following conditions: at 0.00 minutes, the percentage of Solvent B is 20%. This remains the same at 0.60 minutes. At 0.61 minutes, it increases to 50%, and by 1.00 minute, it reaches 100%. The percentage of Solvent B stays at 100% until 1.20 minutes, after which it decreases back to 20% at 1.21 minutes. The eluent was injected directly into an Agilent ToF mass spectrometer (G6230B) using a dual AJS ESI source and scanning between 600-3200 Da with a scan rate of 1.2 s in positive mode. Data acquisition was carried out in 2 GHz Extended Dynamic range mode. Spectra were processed using Mass Hunter Bioconfirm Software 10.0 (Agilent) with the Maximum Entropy method employed. The total ion chromatograms (TIC) were extracted and the summed scans were deconvoluted over a m/z range of 49000-53000 Da.

The deconvoluted spectra were exported as csv files and analyzed using an R script to generate PDF files of the spectra. The median of the protein-only controls were subtracted from the sample spectra to remove baseline signal. The peak height for unmodified protein and protein modified by reactive fragments were recorded and used to calculate percentage modification. In this study, we utilized a synthesis method for WRN inhibitors as described by Ashley Adams et al. 2023, Patent No. WO2023062575 A1(54).

### Covalent modification site identification by tandem MS

GSK_WRN1 (20 µM) was incubated with WRN helicase domain (2 µM) and incubated at 21 °C for 24 hrs. The sample was separated by SDS-PAGE to remove excess unbound compound. Gels were stained with colloidal Comassie InstantBlue, and bands corresponding to WRN were excised, reduced with 10 mM TCEP (65 °C, 30 min), and alkylated with 10 mM iodoacetamide (21 °C, 30 min, dark). Samples were digested with trypsin (Promega) 1:10 E:S (37 °C, 16 hr) in 100 mM ammonium bicarbonate. After the removal of the supernatant, peptides were extracted using acetonitrile. Combined supernatants were concentrated in a SpeedVac centrifuge and acidified (0.1% formic acid, 0.05% trifluoroacetic acid) before injection into the LC-MS/MS system. Digested samples were injected on an Easy-nLC 1000 UHPLC system (Thermo Scientific). The nanoLC was interfaced to a Q-Exactive Hybrid Quadrupole-Orbitrap Mass Spectrometer (Thermo Scientific) and separated on a 25 cm x 75 µm, 2 µm particles, PepMap C18 column (Thermo Scientific) using a 50 min gradient of 2-38% acetonitrile, 0.2% formic acid and a flow rate of 300 nL/min. LC-MS/MS-based peptide sequencing was performed using data-dependent analysis (DDA). Uninterpreted tandem MS spectra were searched for peptide matches against the sequence of WRN helicase domain using Mascot (v2.6.0) software with a 5 ppm mass tolerance for peptide precursors and 20 mDa mass tolerance for fragment ions. Raw files were searched using trypsin as the enzyme with up to 2 missed cleavages, and the variable modifications caramidomethylation on cysteine and oxidation on methionine were allowed. Masses corresponding to GSK_WRN1 were allowed as variable modifications on cysteine. MS/MS spectra were manually validated and annotated.

### WRN unwinding assay

Functional WRN unwinding activity was measured using a fluorogenic plate based 384 well assay configured to measure the separation of labeled double stranded DNA substrate. Compounds were dosed out in neat DMSO with a 1:3 serial dilution scheme. 100 nL of compound was stamped into Greiner low volume black assay plates (Greiner Cat#784076) using the Echo Acoustic Dispenserto generate assay ready plates. All solutions were prepared in assay buffer (25 mM TRIS (pH8.0), 5 mM NaCl, 2 mM MgCh, 1 mM DTT, 0.05% BSA) for this 10 μL low volume reaction. To prepare the solutions, a 2X WRN Enzyme cocktail was made containing 200 pM of recombinant full-length WRN protein (1- 1432). A 2X Substrate cocktail was made to consist ofboth 200 μM ATP (any ultra pure ATP sample) and 12 nM of the fluorescent quenched labeled double stranded DNA oligomer (IDT Custom synthesis; 5'-5IABkFQ (SEQ ID NO. 1)/GCA CTG GCC GTC GTT TTA CGG TCG TGA CT-3' (SEQ ID NO. 2): 5'-TTT TTT ACT TAA CGA CGG CCA GTG C (SEQ ID NO. 3)/36-TAMTSP/-3' (SEQ ID NO. 4)). To start the reaction, 5 μL of assay buffer was added to a single column to serve as the low control. Following this, 5μL of 2X WRN enzyme was added in all wells except the buffer low control wells. The reaction plate was covered and incubated at ambient temperature for 4 hours to allow for time dependent inhibition ifit existed. After 4 hours, the addition of 5 μL of 2X-ATP/DNA substrate cocktail was added across all wells ofthe assay plate. This initiated the reaction, as the plate was incubated at ambient temperature for 60 minutes for the unwinding reaction to occur. A 10 mM EDTA solution was prepared and added at 5 μL across the entire plate after 60 minutes to quench the samples for an endpoint measurement. Fluorescent intensity was measured using excitation and emission wavelengths of 525 nm and 598 nm, respectively. High florescent intensity (DMSO with buffer) represents full inhibition of unwinding activity and low fluorescent intensity (DMSO with enzyme) represents no inhibition of unwinding activity. The potency of the compounds was determined using a four-parameter inhibition model to generate pIC50, Hill Slope and maximum inhibition.

### Whole-genome proteomics

SW48 cells were treated with DMSO or 10µM GSK_WRN2 for 48h in triplicates. For harvesting, the cells were washed in PBS, pelleted, and snap-frozen in liquid N2. Cells were lysed in 4% SDS, DNA was digested by benzonase following dilution to 1% SDS. Lysates were cleared by centrifugation, and the supernatant snap frozen until further processing. Protein digestion, labeling with tandem mass tags (TMT), pooling and pre-fractionation were done as described previously(55). Each of the 12 fractions was measured on an Orbitrap Eclipse Tribrid mass spectrometer (Thermo Scientific) online-coupled to an Ultimate3000 nanoRLSC (Dionex). Peptides were separated on custom-made 50 cm × 100 μm (ID) reversed-phase columns (C18, 1.9 μm, Reprosil-Pur, Dr. Maisch) at 55 °C. Gradient elution was performed from 2% acetonitrile to 40% acetonitrile in 0.1% formic acid and 3.5% DMSO within 60 min. MS settings were as follows: Single compensation voltage FAIMS (FAIMS CV = -45), 60k orbitrap resolution, scan range 375-1200 for the master scan. For data-dependent scans, an isolation window of 0.7, first mass at 100, HCD with a collision energy of 38%, Orbitrap resolution of 15k with enhanced resolution mode TMT and TMTpro reagents, a maximum injection time of 60ms, an AGC target of 100000 and a total cycle time of 1.5 seconds was selected. Dynamic exclusion was enabled. Statistical analysis of expression proteomics was done using the statistical environment R. Proteins were filtered to have more than 1 quantified peptide, and annotated contaminants (based on a previously determined contaminant list) were removed. The log2 ion intensities were quantile normalized and used as a measure for protein abundance. A linear model with an empirical bayes variance moderation, implemented in the limma package(56), was used for differential analysis. The resulting p-values were adjusted for multiple testing using the method of Benjamini and Hochberg (BH). Proteins with an absolute log2 fold change larger than log2(1.5) and with an adjusted p-value below 0.05 were considered to have significantly changed under treatment. To perform gene-set enrichment analysis (GSEA), we utilized the GSEA software obtained from the Broad Institute GSEA portal (http://software.broadinstitute.org/gsea/index.jsp). We conducted a pre-ranked GSEA using FC values, applying the default parameters. To estimate the significance of enrichment, we used 1,000 gene permutations.

### Cysteine profiling

Cysteine profiling was done based on a previously published protocol(36). Jurkat cells were treated either with GSK_WRN4 at 10µM or 50µM, each in triplicate or DMSO in quadruplicate for 4h (2 million cells per well in a 96well plate). For harvesting, cells were washed in PBS, pelleted, and snap-frozen in liquid N2. Cells were lysed in 4% SDS, DNA was digested by benzonase following dilution to 1% SDS. Lysates were adjusted for a protein concentration of 2.5 mg/ml. 20µl lysate were incubated with desthiobiotin iodoacetamide (DBIA) at a final concentration of 500µM for 2h at room temperature. Residual DBIA was quenched with 5 mM DTT at a final concentration for 30 minutes at room temperature. Remaining free cysteines were alkylated with 20 mM iodoacetamide at a final concentration for 30 minutes at room temperature in the dark. Protein were digested, labeled with tandem mass tags (TMT) and pooled as described before(55). Desthiobiotin labeled peptides were enriched by incubation with 25µl neutravidin beads for 1h at 4°C. Beads were washed three times with 2ml 100 mM HEPES and three times with 2 ml distilled H_2_O. Desthiobiotin labeled peptides were eluted by incubation with 250µl 50% acetonitrile 0.1% TFA for 20 minutes while gently shaking. Samples were concentrated in a speedvac until dryness and pre-fractionated into three fractions using AssayMAP 5 µL Reversed Phase (RP-S) cartridges on a BRAVO liquid handling station according to manufacturers instructions. Samples were measured on an Orbitrap Exploris mass spectrometer (Thermo Scientific) as described before(55). Peptide and protein identification was done as described previously except for adding the masses of DTB-IAA on cysteine (296.184841) and on Selenocysteine (296.184841) as variable modifications (55). Analysis was done on peptide level filtered for (1% FDR) and the same quantification thresholds as described before(55). Peptides were filtered for presence of the DTB-IAA mass tag on cysteine or selenocysteine. A two-sided t-test determined significance. Peptides with a p-value below 0.001 and an absolute mean log2 fold change of log2(1.5) were considered significantly affected.

### Cell growth inhibition studies (washout experiments)

SW48 and SW620 were cultured with 10% FBS (SAFC Sigma # 12176C-1000mL) in RPMI Medium 1640 medium (Gibco #22400-071) and grown at 37 °C and 5% CO2 air incubator. Cells were trypsinized and counted, seeded at 500 cells per well in 100μL culture medium in a 96-well cell culture assay plate. Compounds were diluted 1:2 from 30μM and added to 96-well plates when cells attached (24 hours post seeding). At different timepoints (1.5, 3, 5, 7, 24, 48, 72 hours) after compound addition, culture media with compounds were aspirated. Cells were washed with 1X warm PBS gently and replaced with 100 μL fresh media. Cells were cultured for a total duration of 6 days from compound addition. Cell viability readout is performed with CellTiter-Glo One kit (Promega #G8462) and measured on an Envision Multiplate reader (PerkinElmer). Luminescence data was normalized to DMSO-treated wells, and dose-response curve fitting and plotting was performed in GraphPad Prism 7.0.

### Animal studies

All animal handling, care, and treatment procedures in this study adhered to the guidelines approved by the Institutional Animal Care and Use Committee (IACUC) and followed the Association for Assessment and Accreditation of Laboratory Animal Care (AAALAC) standards. The in vivo study was performed in accordance with the GSK 3Rs (Replacement, Reduction, Refinement) principle. Experimental protocols for SW48, SW620, and Patient-Derived Xenografts (PDX) received approval from the IACUC of GSK and were conducted at the GSK US Cambridge site. LS411N and HT29 protocols received approval from the IACUC of Crown Bioscience and were carried out at the Beijing site of CrownBio Science.

For the xenografting process, 5x10^6 cells each of SW48 and SW620 were mixed in a 1:1 ratio with RPMI and Matrigel (100 µL) and then xenografted into the right flank of Crl:Nu-Foxn1 nu (Strain# 088, Charles River Laboratory, Wilmington, MA USA) female mice, aged 6-8 weeks and weighing 20-25g. Similarly, 3x10^6 cells each of LS411N and HT29 were mixed 1:1 with PBS and Matrigel or PBS alone (100 µL) and xenografted into the right flank of female BALB/c Nude mice (GemPharmatech Co. Ltd, China), aged 6-7 weeks. PDX samples, sized 3mm x 3mm, were implanted using a 10G Trocar into the right hind flank of NOD.Cg-prkdcscid IL2rgtmWjl/Szj mice (Strain # 005557, Jackson Lab, Bar Harbor, ME USA), aged 6-8 weeks and weighing over 17g.

Once the tumors reached approximately 80-140 mm^3^, animals were randomly assigned into groups based on individual tumor size using a stratified for SW48, SW620, and PDX and matched distribution for LS411N and HT29 randomization method. Body weight and tumor volume were measured twice per week using an electric caliper (Fowler Ultra Cal V) and an Ohaus electronic scale (STX421), with data automatically recorded in the Study Log. Tumor volume was calculated using the formula 0.5 x L x W^2, where L is the tumor length (the longest dimension) and W is the tumor width (perpendicular to L). Tumor growth inhibition was calculated using the following equation: Mean % Δ Inhibition = [(mean(C)-mean (C0)) - (mean (T)-mean (T0))] / [mean (C)- mean (C0)] * 100%.

For pharmacodynamic analysis of GSK_WRN4, tumors were collected at different time point 24 hours after the final dose. Tumor tissues were divided into two portions: one for snap freezing and the other fixed in 10% formalin for 24 hours and then embedded in paraffin for histological analysis. GSK_WRN4 was freshly prepared using 20% propylene glycol, and animals received daily oral administration of GSK_WRN4 at a selected dose with a volume of 10 mL/kg. Statistical analyses were performed using two-way ANOVA to compare control vs. GSK_WRN4 treatment at different time points and relative body weight (as a percentage of Day 0, between control and GSK_WRN4 at different time points) using Prism GraphPad Prism 9 (GraphPad Software, Inc., San Diego, California, USA). All data are presented as mean ± standard error. Statistical significance was determined by main effects found via Tukey’s post hoc test, with a p-value of less than 0.05 considered statistically significant. For pharmacokinetic studies, GSK_WRN4 was orally administered in the male BALB/c mouse as a suspension formulation. Two mice were used for the PK study under fed conditions. The compound was formulated in 1% aqueous methyl cellulose, in a white fluid suspension and administered through oral gavage. The blood of mice was sampled via tail snip method at 0, 0.25, 0.5, 1.5, 2, 4, and 8 hours after dosing. 25uL blood from each sample was used for LC/MS/MS analysis of blood concentrations of GSK_WRN4.

### In vivo tumor histological staining

All histological analyses were performed by Histowiz, Inc. (Brooklyn, New York, USA). Standard procedures for Hematoxylin and Eosin (H&E) staining, Masson's Trichrome staining, and Immunohistochemistry (IHC) were utilized to assess morphology, fibrosis/extracellular matrix composition, and specific protein expression within the tissue samples, respectively. For the IHC, the following primary antibodies were employed: Ki67 (ab15580, 1:800, Abcam, Waltham, MA, USA), Phospho-Histone H2A.X (γH2AX) (CST9718, 1:800, Cell Signaling Technology, Danvers, MA, USA), Phospho-KAP1 (S824) (ab2438570, 1:1000, Abcam, Waltham, MA, USA), p21 (ab109520, 1:100, Abcam, Waltham, MA, USA), and Cleaved Caspase 3 (CST9661, 1:300, Cell Signaling Technology, Danvers, MA, USA).

### Validation experiments in the independent cohort of CRC organoids

The Colon cancer patient derived organoid screening was performed by CrownBio Science (Beijing, China). A thorough examination was conducted using a comprehensive panel consisting exclusively of GSK_WRN4, which was applied to the organoids. To evaluate the effects of the treatment, a 5-day CellTiter-Glo Luminescent Cell Viability Assay was employed, which assessed the viability changes occurring in the organoids in response to the GSK_WRN4. Deficiencies in the mismatch repair system were evaluated using an MMR panel test through IHC. This test specifically assessed the expression of MLH1, MSH2, MSH6, and PMS2. The analysis was conducted at NeoGenomics Laboratories (Fort Myers, FL, USA).

### Data analysis

Heatmaps in the biomarker analysis section were created using the geom_tile function from the ggplot2 package in R. The plots and graphs were generated using GraphPad and Spotfire software. The WRN dependency for cell lines was determined from an integrated dataset of essentiality data, curated from multiple sources including Project Score (https://score.depmap.sanger.ac.uk/) and the Dependency Map (DepMap; https://depmap.org/portal/), as recently described(57). The analysis in Fig. 3C, including the calculation of Pearson correlation, p-values, and Benjamini-Hochberg (BH) adjusted q-values, was conducted using Python's numpy and stats libraries.

## Supplementary Material

Supplementary figures

Table S1

Table S2

Table S3

## Data Availability

The data generated in this study are available within the article and its supplementary data files. TrAEL-seq sequencing data have been deposited in the Gene Expression Omnibus (GEO) repository under the accession number GSE253197. Base editing sequencing data have been deposited in the EGA database under accession number EGAD00001015355.

## Significance

We report the discovery and characterization of potent, selective WRN helicase inhibitors for MSI cancer treatment, with biomarker analysis and evaluation of efficacy *in vivo* and in immunotherapy-refractory preclinical models. These findings pave the way to translate WRN inhibition into MSI cancer therapies and provide tools to investigate WRN biology.
