# Supplementary material for: Novel WRN Helicase Inhibitors Selectively Target Microsatellite Unstable Cancer Cells
Source: Cancer Discov. Author manuscript; Available in PMC 2024 Nov 26. (PMC7616858; doi:10.1158/2159-8290.CD-24-0052)
Supplement: Supplementary figures [file EMS195351-supplement-Supplementary_figures.docx]

# Supplementary Material

**Novel WRN Helicase Inhibitors Selectively Target Microsatellite Unstable Cancer Cells.**

Gabriele Picco^1*^, Yanhua Rao^2^, Angham Al Saedi^1^, Yang Lee^2^, Sara F. Vieira^1^, Shriram Bhosle^1^, Kieron May^6^, Carmen Herranz-Ors^1^, Samantha J. Walker^1^, Raynold Shenje^2^, Cansu Dincer^1^, Freddy Gibson^1^, Ruby Banerjee^1^, Zoe Hewitson^1^, Thilo Werner^4^, Joshua E. Cottom^2^, Yang Peng^2^, Nanhua Deng^5^, Youyou Zhang^2^, Eldridge Nartey^2^, Leng Nickels^2^, Philip Landis^2^, Daniela Conticelli^7^, Katrina McCarten^1^, Jacob Bush^3^, Mamta Sharma^1^, Howard Lightfoot^1^, David House^3^, Emma Milford^3^, Emma K. Grant^3^, Michal P. Glogowski^2^, Craig D. Wagner^2^, Marcus Bantscheff^4^, Anna Rutkowska-Klute^4^, Cell Model Network UK Group, Francesca Zappacosta^2^, Jonathan Pettinger^3^, Syd Barthorpe^1^, H. Christian Eberl^4^, Brian T. Jones^8^, Jessica L. Schneck^2^, Dennis J. Murphy^2^, Emile E. Voest^9^, Joshua P. Taygerly^8^, Michael P. DeMartino^2^, Matthew A. Coelho^1^, Jonathan Houseley^6^, Geeta Sharma^5^, Benjamin Schwartz^2^ and Mathew J. Garnett^1*^

**Supplementary Figures**

**Supplementary Figure 1**

**
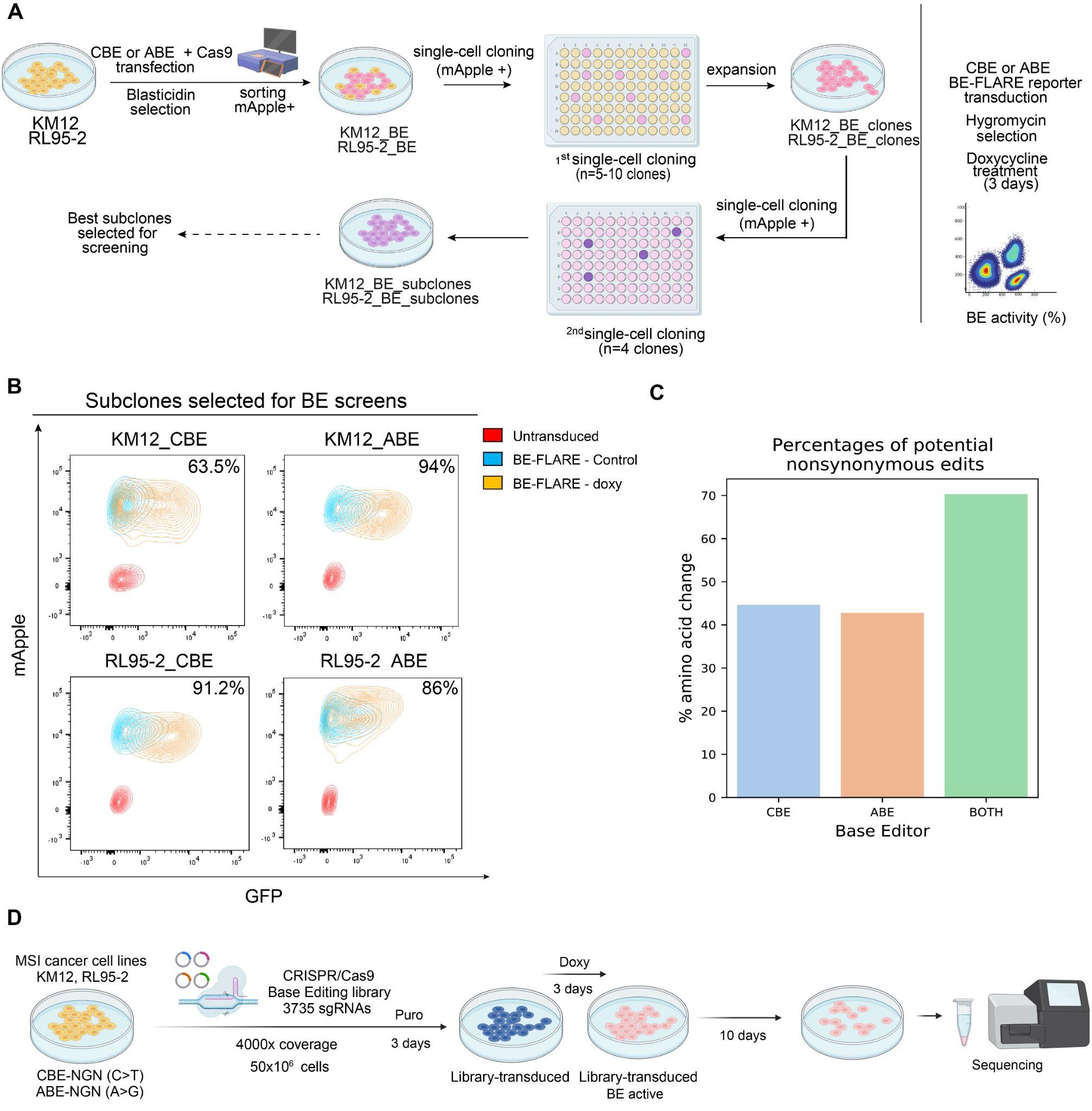
**

***Supplementary Figure 1. Generating base editor-expressing MSI cell lines for screening.*** *(A) Workflow for generating doxycycline-inducible ABE and CBE KM12 and RL95-2 cells. Parental cells transduced with base editor constructs were sorted for mApple+ cells. Cells were transduced with a BE-FLARE reporter before and after subcloning and selected with hygromycin. Base editing activity was measured by GFP induction with doxycycline. After two rounds of subcloning, clones with high editing activity and low leakiness were selected. (B) Quantifying base editing activity in selected KM12 and RL95-2 ABE and CBE subclones by measuring GFP+ cells after doxycycline induction. Plots and percentages show a representative experiment performed in duplicate. (C) Percentage of amino acids in WRN with a predicted non-synonymous edit from sgRNAs in the BE screening library. (D) Schematic of base editing screening strategy in MSI cell lines (created with BioRender.com).*

**Supplementary Figure 2**

*2
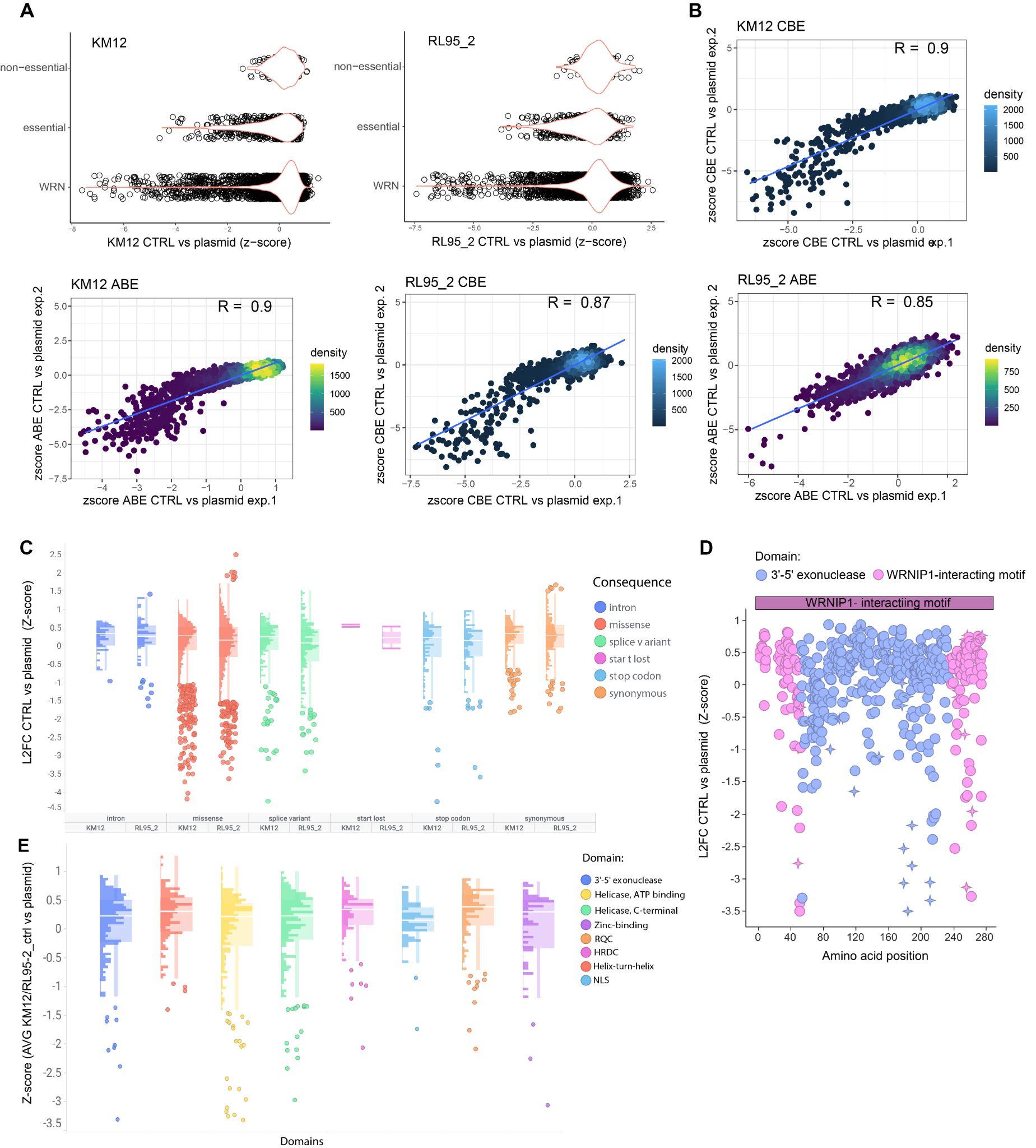
*

***Supplementary Figure 2. Quality control for base-editor screens in MSI cell lines.*** *(A) Density plots of ABE and CBE screens displaying Z-scores for sgRNAs targeting predicted splice variants of essential and non-essential genes or WRN-targeting sgRNAs (control vs. plasmid arm). (B) Replicate correlation for ABE and CBE screens based on sgRNA log2 fold-changes (control vs. plasmid arm) in KM12 and RL95-2. (C) Z-scores (control vs. plasmid) for WRN-targeting sgRNAs in KM12 and RL95-2, stratified by predicted edit consequence. (D) Z-scores (control vs. plasmid) for WRN-targeting sgRNAs in KM12. The Exonuclease domain and WRNIP1 interacting motif are highlighted. Cross symbols mark the predicted edits likely to result in the insertion of proline residues. (E) Average Z-score distribution from the base editing screens in the two cell lines, subdivided by WRN functional domains. Only missense mutations not predicted to install proline amino acid were included in this graph.*

**Supplementary Figure 3**

**
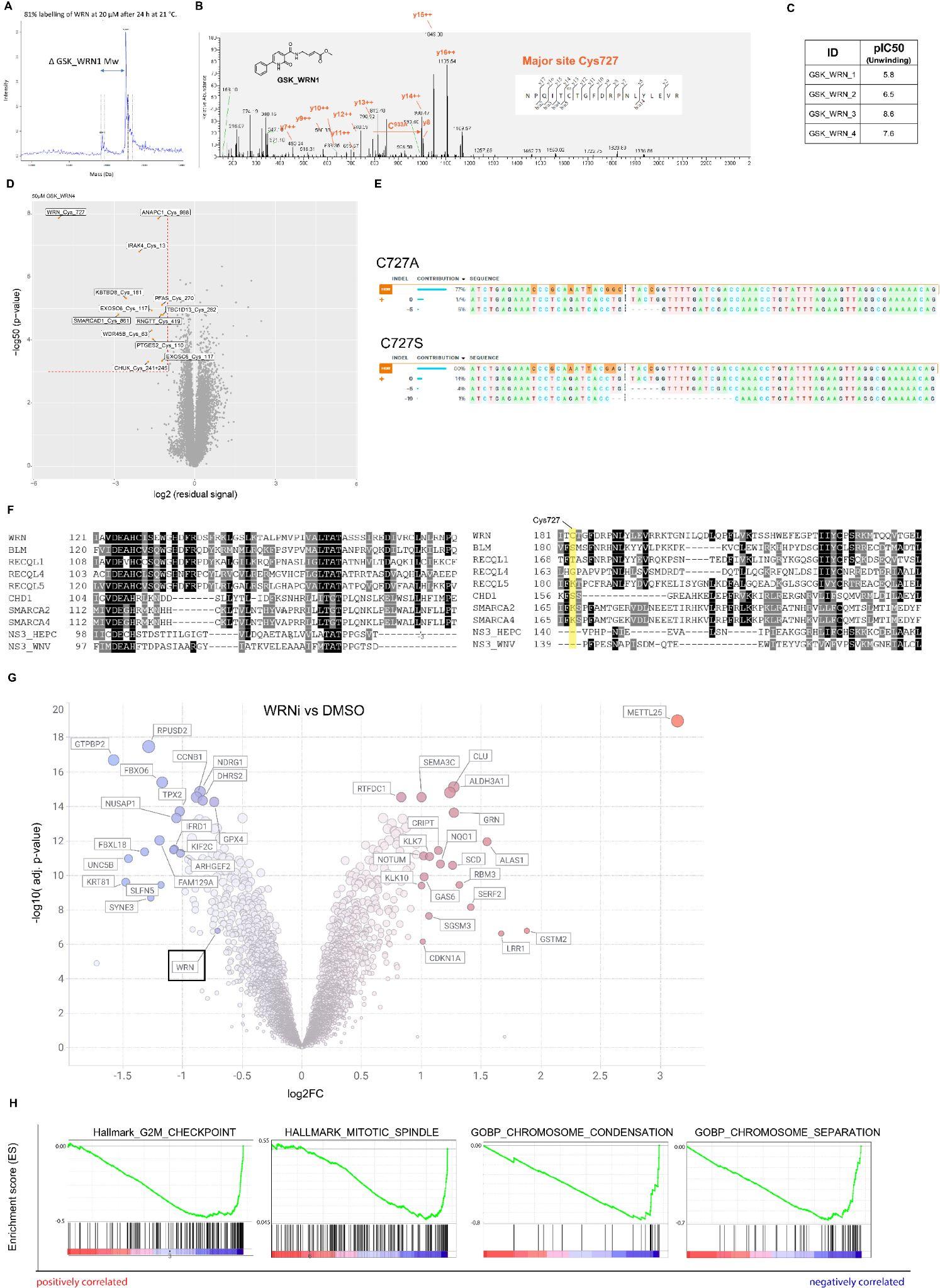
**

***Supplementary Figure 3. Discovery and characterization of novel inhibitors of WRN helicase*** *(A) Intact-protein LCMS trace showing WRN helicase domain covalently modified by GSK_WRN1. (B)Tryptic digest LC-MS/MS spectra showing covalent modification of Cys727 by GSK_WRN1. (C) The table displays the inhibitory effects of GSK_WRN compounds (1-4) on WRN helicase activity, measured as pIC50 values (the logarithmic concentration needed to achieve 50% inhibition) from the human WRN full-length unwinding assay. The assay was conducted over 4 hours using the FLINT method for endpoint inhibition analysis. Higher pIC50 values indicate stronger inhibition. (D) Volcano plot of quantitative reactive cysteine profiling of Jurkat cells treated with 50 μM GSK_WRN4. Most strongly regulated proteins and the target cysteine residue WRN Cys727 are indicated. (E) CRISPR-Cas9 editing verification in SW48 isogenic C727A/S cells. Displays guide RNA target, PAM sequence, indel frequency, model fit, and knock-in score. (F) RecA and RecQ helicase proteins are aligned around Cys727 (number Cys183 in alignment, highlighted in yellow; the amino acid number is from the helicase domain only). Cysteine is not present in any other family proteins. (G) Volcano plot of expression proteomics analysis of SW48 cell line treated with 10 µM GSK_WRN2 for 48h (n=3) compared to DMSO control (n=3). The names of the most strongly regulated proteins and the pharmacological target WRN are indicated. (H) representative GSEA gene set enrichment results for whole-genome proteomic analysis performed in MSI SW48 cell lines treated with GSK_WRN2 compared to DMSO control.*

**Supplementary Figure 4**

*
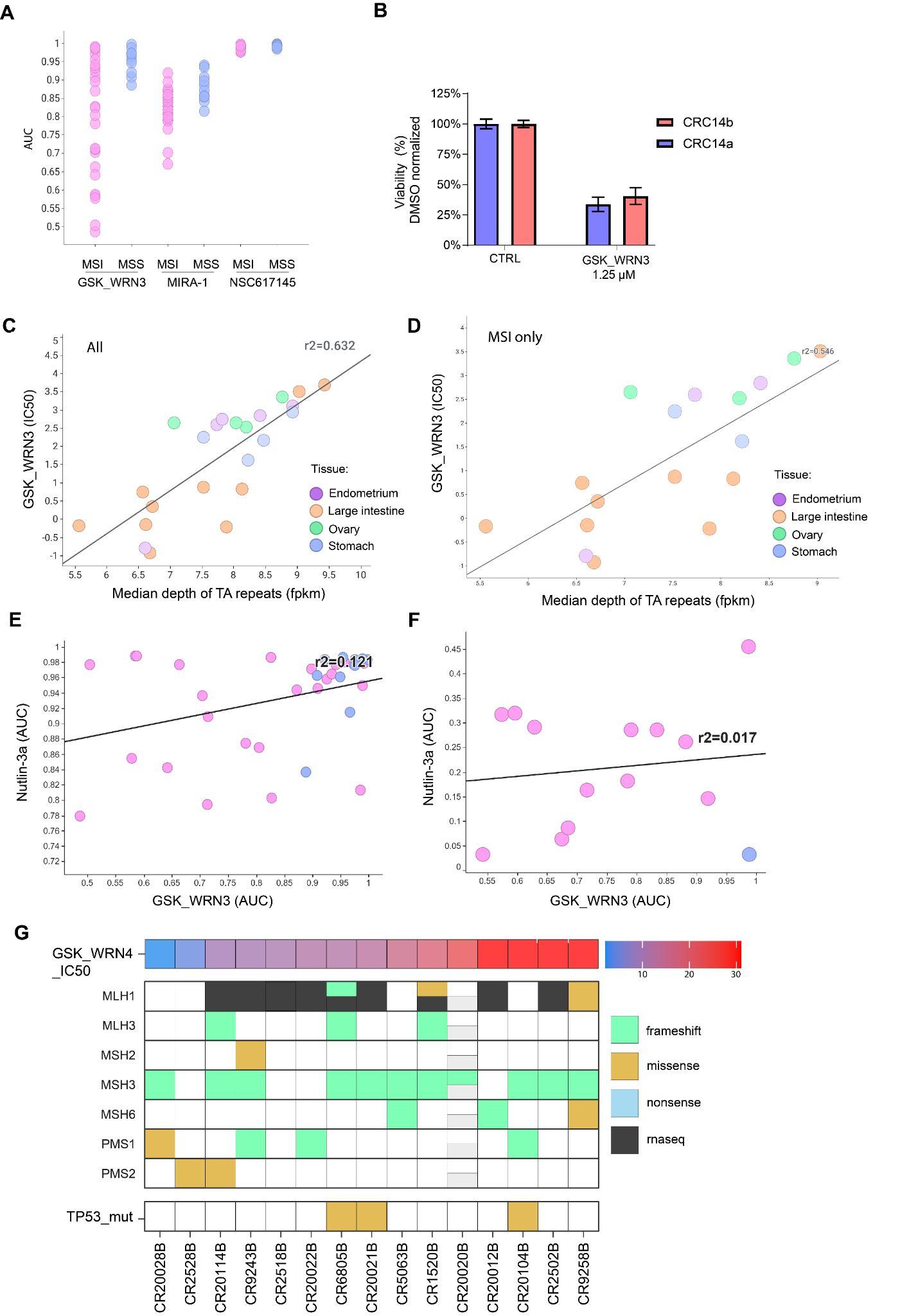
*

***Supplementary Figure 4. Correlation of WRN inhibitor sensitivity with genetic and molecular markers in MSI models.*** *(A) Dot plot depicting the distribution of area under dose-response curve (AUC) values for WRN inhibition in MSI and MSS cell lines treated with GSK_WRN3, MIRA-1, and NSC617145. (B) Cell viability assay comparing the effects of GSK_WRN3 (1.25 μM) on CRC14b and CRC14a organoids. The control (CTRL) group was only treated with DMSO. Data normalized to DMSO control and expressed as percentage viability. Error bars represent the standard deviation of three technical replicates. (C) Correlation between the IC50 of GSK_WRN3 and the median sequencing depth of 'broken' TA repeats, determined by coverage analysis from whole genome sequencing data. The data represent a broad range of cell lines from MSI-predominant lineages. The correlation coefficient (r^2) signifies the strength of this association. (D) Same as C but only MSI cell lines. (E) and (F) Scatter plots demonstrating the correlation between AUC values for Nutlin-3A and WRN inhibitor GSK_WRN3 across MSI and MSS cell lines for MSI-predominant lineages (E) and CRC organoids (F). The correlation coefficient (r^2) is provided for each comparison, indicating the degree of linear relationship between the sensitivities to different inhibitors. (G) Heatmap and corresponding mutation status grid for CRC patient-derived organoids treated with GSK_WRN4. The heatmap illustrates GSK_WRN4 IC50 values, with warmer colors indicating higher IC50 (reduced sensitivity). Below, a grid shows the mutation status of MMR-pathway genes (MLH1, MLH3, MSH2, MSH3, MSH6, PMS1, PMS2) and TP53, coded by mutation type: frameshift, missense, nonsense, and confirmed by RNAseq. The organoids are categorized by tissue origin, indicated by color-coded bars at the bottom of the heatmap.*

**Supplementary Figure 5**

*
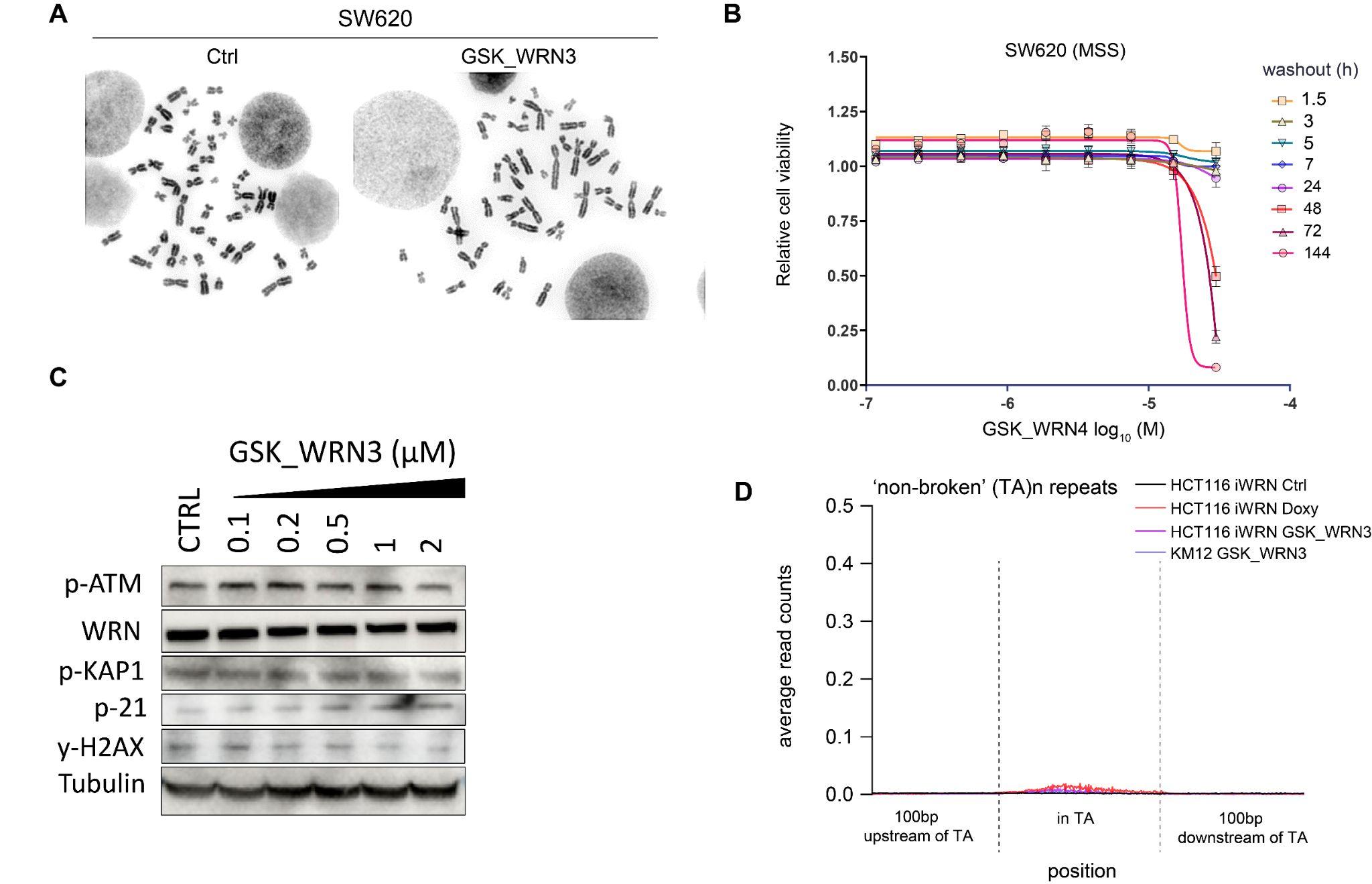
*

***Supplementary Figure 5. Comparative analysis of DNA damage in MSI and MSS cells treated with WRN inhibitors and genetic inactivation*** *(A) Representative images of SW620 (MSS) metaphase spreads harvested after 24h treatment with DMSO-control or GSK_WRN3. (B) Time- and dose-dependent inhibition of SW620 cell growth by compound GSK_WRN4. SW620 cells were treated with 0.1-20 μM for 1.5, 6, 12, 24, 48, 72, or 144 hours. GSK_WRN4 was then washed out, and cell growth was assessed by cell counting over 72 hours. Data points represent the mean ± SD of three independent experiments. (C) Immunoblot of phospho-ATM, WRN, γ-H2AX, phospho-KAP1 and p21 following treatment with GSK_WRN3 in SW620. (D) Metaplots display TrAEL-seq signal for 'non-broken' TA repeats in an HCT116 c9 clone. This clone is characterized by the expression of sgRNA targeting WRN, which is under inducible control. The figure illustrates the variations in TRAEL-seq signal under different conditions: treatment with DMSO (control), doxycycline (to induce sgRNA expression), and GSK_WRN3. Additionally, the effect of GSK_WRN3 treatment on KM12 cells is also shown. The Y-axis represents the average read counts of the peaks, while the X-axis is segmented into three parts indicating the position of the peaks: 100bp upstream, directly within, and 100bp downstream of the TA repeat site.*

**Supplementary Figure 6**

***
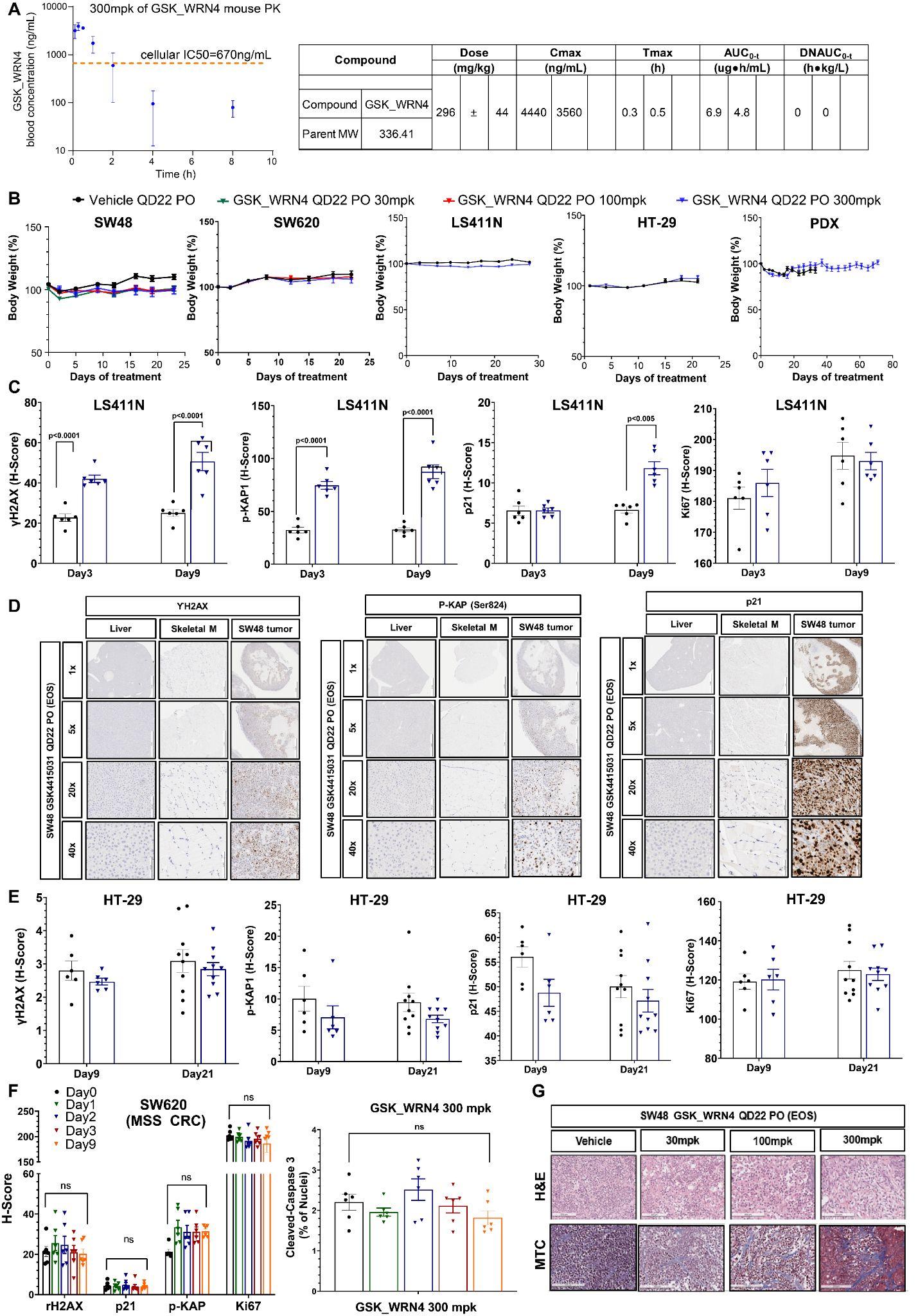
***

***Supplementary Figure 6: Effects of GSK_WRN4 on MSI and MSS colorectal cancer xenografts.***

*(A) The graph depicts the plasma concentration of GSK_WRN4 over time following a single oral dose in a mouse model. The blue diamonds mark the mean concentration at each time point, while the dashed orange line represents the desired therapeutic level. Error bars show the standard deviation, illustrating the range of responses at each time interval. The table reports data from individual animals. Cmax = maximum concentration; Tmax = time Cmax is achieved; AUC0-t = area under the plasma concentration-time curve to the last time point with quantifiable drug; DNAUC0-t = dose‑normalized AUC0-t. (B) Body weight trajectories of mice bearing xenografts derived from MSI cell lines SW48 and LS411N, MSS cell line SW620 and HT29, and an MSI patient-derived xenograft (PDX) were monitored during treatment with GSK_WRN4, indicating stable body weight across various dosages throughout the experiment period. (C) Quantification of DNA damage markers γH2AX, p-KAP1, Ki67 and p21 in LS411N xenografts at Day 3 and Day 9 of GSK_WRN4 treatment, demonstrating dose-responsive increases. (D) Immunohistochemical analysis for γH2AX, p-KAP1, and KI67 in SW48 tumor sections compared to liver and skeletal muscle from same animal, showing targeted DNA damage is specific in human MSI-H tumor via WRN helicase, rather than being an off-target effect. (E) Quantifying γH2AX, p-KAP1, p21, and KI67 in MSS HT-29 xenografts confirms the absence of DNA damage after GSK_WRN4 treatment. (F) Quantification of DNA damage markers in SW620 MSS colorectal cancer xenografts, confirming the specificity of GSK_WRN4 for MSI over MSS models. (G, top panel) Hematoxylin and eosin (H&E) staining of representative SW48 tumors treated with GSK_WRN4, illustrating changes in tumor cellularity and nuclear morphology. (F, bottom panel) Masson's trichrome staining of representative SW48 tumors, highlighting modifications in stromal elements and extracellular matrix composition.*
